# Isolation and Characterization of Antimicrobial Peptides with Unusual Disulfide Connectivity from the Colonial Ascidian *Synoicum turgens*

**DOI:** 10.3390/md18010051

**Published:** 2020-01-12

**Authors:** Ida K. Ø. Hansen, Johan Isaksson, Aaron G. Poth, Kine Ø. Hansen, Aaron J. C. Andersen, Céline S. M. Richard, Hans-Matti Blencke, Klara Stensvåg, David J. Craik, Tor Haug

**Affiliations:** 1 Norwegian College of Fishery Science, Faculty of Biosciences, Fisheries and Economics, UiT The Arctic University of Norway, Breivika, N-9037 Tromsø, Norwayceline.s.richard@uit.no (C.S.M.R.); hans-matti.blencke@uit.no (H.-M.B.); klara.stensvag@uit.no (K.S.); 2 Department of Chemistry, UiT The Arctic University of Norway, Breivika, N-9037 Tromsø, Norway; johan.isaksson@uit.no; 3 Institute for Molecular Bioscience, The University of Queensland, Brisbane 4072, Queensland, Australia; a.poth@imb.uq.edu.au (A.G.P.); d.craik@imb.uq.edu.au (D.J.C.); 4 Marbio, UiT The Arctic University of Norway, Breivika, N-9037, Tromsø, Norway; kine.o.hanssen@uit.no

**Keywords:** marine, ascidian, peptide, antimicrobial, methionine oxidation

## Abstract

This study reports the isolation of two novel cysteine-rich antibacterial peptides, turgencin A and turgencin B, along with their oxidized derivatives, from the Arctic marine colonial ascidian *Synoicum turgens*. The peptides are post-translationally modified, containing six cysteines with an unusual disulfide connectivity of Cys^1^-Cys^6^, Cys^2^-Cys^5^, and Cys^3^-Cys^4^ and an amidated C-terminus. Furthermore, the peptides contain methionine residues resulting in the isolation of peptides with different degrees of oxidation. The most potent peptide, turgencin A_Mox1_ with one oxidized methionine, displayed antimicrobial activity against both Gram-negative and Gram-positive bacteria with a minimum inhibitory concentration (MIC) as low as 0.4 µM against selected bacterial strains. In addition, the peptide inhibited the growth of the melanoma cancer cell line A2058 (IC_50_ = 1.4 µM) and the human fibroblast cell line MRC-5 (IC_50_ = 4.8 µM). The results from this study show that natural peptides isolated from marine tunicates have the potential to be promising drug leads.

## 1. Introduction

The emergence of pathogenic microorganisms’ resistance to conventional antibiotics has become a serious medical concern [1]. This natural microbial adaptation strategy [2] has been provoked by selective pressure exerted by extensive inappropriate use of antimicrobial agents, both for medical and non-medical purposes [3]. Diminished efforts in the development of novel antimicrobials by the pharmaceutical industry have further aggravated this problem [4,5]. Renewed efforts in the search for novel forms of antimicrobial treatments are now seen. Endogenous antimicrobial peptides (AMPs) are considered exciting candidates to address this challenge due to their innate properties of broad antimicrobial spectra, highly selective toxicities, and the increased difficulty for microbes in developing resistance towards them compared to conventional small molecule antimicrobial agents [6].

AMPs (also called host defense peptides, HDPs) are ancient effector molecules of the innate defense system, and are present in every life form [7,8]. Their evolutionary conservation among eukaryotes highlights their importance in the first-line of defense against invading pathogens [9]. They generally consist of 10 to 50 amino acids, have an amphiphilic three-dimensional structure and a net positive charge at physiological pH [7]. AMPs are arranged into highly heterogeneous structures [6], grouped into linear α-helical peptides, β-sheets stabilized by intramolecular disulfide bridges, and extended structures [10]. Unlike conventional antibiotics, which typically target specific cellular pathways necessary for microbial survival or reproduction [11], it is widely accepted that most AMPs exert their effect by targeting and destabilizing the lipopolysaccharide layer of the cell membrane, which is ubiquitous in microorganisms. The cationic AMP portion targets the anionic microbial membrane through unspecific electrostatic interaction. This is followed by membrane embedding of the hydrophobic portion, causing membrane disruption and cell death [12,13,14]. This non-specific targeting of an essential microbial component lowers the chance of resistance development towards AMPs, for which the bacteria need to change the entire membrane lipid composition, a costly solution for most microbes [9]. In addition, AMPs at large do not react with the lipophilic outer leaflet of mammalian cell membranes, hence their low tendency to be toxic towards human cells [15]. However, AMPs with antitumor activities have also been isolated [16].

The oceans cover 71% of the earth’s surface and comprise 50–80% of the total global biodiversity [17]. Many marine invertebrates are sessile and soft bodied, lacking the sophisticated adaptable immune systems seen in vertebrates. Despite this, these organisms thrive, suggesting that their innate immune systems are effective and robust. It is now known that this apparent contradiction can be explained by the activities exerted by AMPs [18]. Compared to terrestrial AMPs, marine-derived AMPs are often adapted to high salt concentration conditions, enabling them to form stronger electrostatic interactions with bacterial membranes, thus making marine-derived AMPs more potent [19,20]. AMPs have been found in a wide range of marine invertebrates, including mollusks [21], crustaceans [22], sponges [23], and cnidarians [24]. A number of α-helical AMPs have also been isolated from a number of ascidians (belonging to the subphylum *Urochordata*, bearing all the chordate hallmarks in its larval form), including the phenylalanine-rich styelins and the histidine-rich clavanins from *Styleaclava* [25,26] and halocynthin and papillosin from *Halocynthia papillosa* [27]. Furthermore, two cysteine-containing α-helical AMPs have been isolated from *H. aurantium*: the homodimer dicynthaurin [28] and the heterodimer halocidin [29].

As part of our ongoing search for new compounds with antimicrobial activity from marine invertebrates, the aqueous extract of the colonial ascidian *Synoicum turgens* was examined for its content of AMPs. *Synoicum* species have previously awarded several bioactive secondary metabolites, including the cytotoxic palmerolide macrolides [30,31], the β-carboline guanidine alkaloid tiruchanduramine with α-glucosidase inhibitory activity [32,33], and the synoxazolidinones and pulmonarines with various bioactivities from *S. pulmonaria* [34,35,36,37]. To the best of our knowledge, no AMPs have been isolated and characterized from *Synoicum* species.

In the present study, we isolated, purified and characterized four novel AMPs, named turgencin A_Mox1_, turgencin B, turgencin B_Mox1_, and turgencin B_Mox2_ from the Arctic ascidian *S. turgens*. The peptides were screened for antimicrobial activity against both Gram-negative and Gram-positive bacterial strains, and for cytotoxic activities against selected human cell lines. The goal of this study was to find new peptides with a potential for treating microbial infections.

## 2. Results and Discussion

### 2.1. Peptide Purification and Mass Spectrometry Analysis

**Figure 1:**
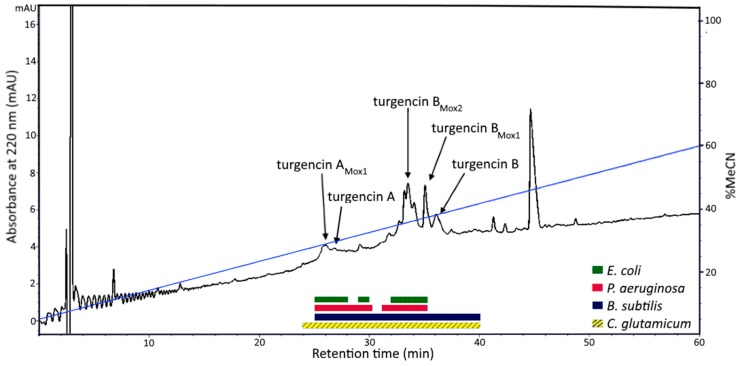
Preparative RP-HPLC-DAD spectrum at 220 nm of the 80% MeCN SPE eluate of *S. turgens*. HPLC fractions displaying antibacterial activity are shown in boxes below the chromatogram and peak fractions containing antibacterial peptides are marked with arrows. The blue line shows the linear gradient (5-60%) of acetonitrile (MeCN) dissolved with 0.1% formic acid (FA). Note: RP— reversed-phase; HPLC—high performance liquid chromatography; DAD—diode array detector; SPE—solid phase extraction.

Solvent extraction of the *S. turgens* specimens using 60% (v/v) acetonitrile (MeCN) at low temperature produced two liquid phases, an MeCN-rich organic phase and a salt-rich aqueous phase. This separation is caused by the high salt content within marine samples, which is immiscible with MeCN at low temperature [38]. Salt was subsequently removed from the aqueous extract using solid phase extraction (SPE), yielding eluates with compounds of varying polarity. Antimicrobial screening of the SPE eluates and the organic extract revealed that the 40 and 80% MeCN SPE eluates displayed the highest antibacterial activity (Table S1 in the SI). The 80% MeCN SPE eluate was further fractionated by preparative reversed-phase high performance liquid chromatography (RP-HPLC), guided by a diode array detector (DAD) measuring UV-vis absorption at 220 and 280 nm. The obtained fractions were tested for antibacterial activity and the active fractions were analyzed by ultra-high performance liquid chromatography coupled to a quadrupole time-of-flight mass spectrometer (UHPLC-QTOF-MS). Several HPLC fractions displayed antibacterial activity and four bioactive peptides were extensively purified by RP-HPLC (Figure 1). The four peptides were also shown to be present in the 40% MeCN SPE eluate, but in lower quantities (data not shown). The peptides were present in all *S. turgens* samples regardless of where and when the biomass was collected.

The four isolated peptides (turgencin A_Mox1_, turgencin B, turgencin B_Mox1_, turgencin B_Mox2_) were analyzed by UHPLC-QToF-MS to determine their monoisotopic molecular masses and isotope patterns, and to provide fragmentation ions in tandem mass spectrometry (MS/MS) (Figure S1 in the SI). This analysis resulted in the determination of monoisotopic masses of 3705.79, 3538.58, 3554.58, and 3570.58 Da, for turgencin A_Mox1_, turgencin B, turgencin B_Mox1_, turgencin B_Mox2_, respectively (Table S2 in the SI).

### 2.2. Sequence Analysis

The peptides turgencin A_Mox1_ and turgencin B_Mox2_ were subjected to reduction/alkylation and N-terminal Edman degradation sequencing. A partial N-terminal sequence (34 residues: GPKTKAACKMACKLATCGKKPGGWKCKLCELGCD) was obtained for turgencin A_Mox1_. The calculated monoisotopic mass of this sequence (3520.68 Da, assuming three disulfide bridges) was determined to be 185.11 Da less than the measured monoisotopic mass, indicating a missing dipeptide C-terminally. Reduction and alkylation with iodoacetamide, followed by enzymatic digestion with Glu-C, RP-HPLC, and Edman degradation sequencing of selected HPLC fractions produced the fragment LGCDAV, which represents the C-terminal sequence from position 31 to 36. MS analysis proved this fragment (*m/z* 633.29, corresponding to [M+H]^+^ and containing one carboxyamidomethylcysteine) to be amidated C-terminally. The monoisotopic mass of the 36-residue sequence (3689.80 Da, assuming three disulfide bridges and a C-terminally amidated valine) was determined to be 16 Da above the measured monoisotopic mass. This can be explained by an oxidized methionine (Met-ox) residue in position 10 (+15.9949 amu for monoisotopic oxygen). Extracted ion chromatograms of the SPE eluates proved the presence of a peptide with a monoisotopic mass of 3689.80 Da, also indicating the presence of the non-oxidized version of the peptide, turgencin A. By comparing MS/MS data of turgencin A and turgencin A_Mox1_, in addition to the mass difference of 16 Da between them, it could be concluded that turgencin A is the non-oxidized version of turgencin A_Mox1_ (Figure S2 in the SI).

Edman degradation analysis of turgencin B_Mox2_ produced a 35 residue N-terminal sequence (GIKEMLCNMACAQTVCKKSGGPLCDTCQAACKALG) containing six cysteines and two methionine residues. The calculated monoisotopic mass of this sequence (3539.61 Da, assuming three disulfide bridges) was 30.96 Da greater than the measured monoisotopic mass. This can be explained by two Met-ox residues (+31.99 Da) and a C-terminally amidated glycine (−0.98 Da). MS/MS analysis of turgencin B and turgencin B_Mox1_ confirmed that these peptides are non-oxidized and mono-oxidized variants of turgencin B, respectively. The analysis also showed that the mono-oxidized peptide was mainly oxidized at the methionine residue in position 5 (Figure S3 in the SI).

The retention times of both turgencin A, turgencin B, and their oxidized derivatives also correspond with their oxidation states (Figure S4 in the SI), as oxidation of methionine has been shown to decrease peptide retention time in RP-HPLC [39].

**Figure 2:**
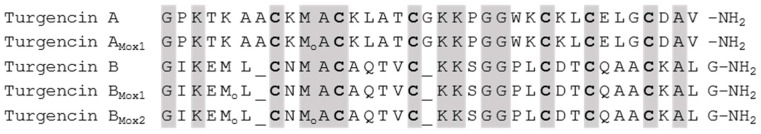
Sequence alignment of turgencin A and B, and their oxidized derivatives. Gaps (_) are introduced to maximize the alignment. Cysteine residues are marked in bold, oxidized methionine residues are marked with M_o_, and identical amino acids are shaded in grey.

Although the primary sequence and peptide length differ between the two mature peptides (Figure 2), there are some similarities. Both peptides contain six cysteine residues with the same cysteine pattern (C-C-C-C-C-C, i.e. no adjacent cysteines) and two identical sequence motifs (CXMAC and KKXGG), indicating identical cysteine connectivity. The peptides are both C-terminally amidated, and cationic with isoelectric points (pI) of 9.24 (turgencin A) and 8.33 (turgencin B). The peptides were not identified to belong to any of the 45 major AMP families present in CAMP_R3_ database using the CAMPSign tool [40]. Furthermore, the basic local alignment search tool (BLAST) and homology searches of the oligopeptide sequences resulted in no overall sequence similarity to other known peptides or proteins. The peptides were therefore considered as novel antimicrobial peptides and named turgencins after the species from which they were isolated.

Peptides with Met-ox residues (+16/32 Da) could be observed both with MS/MS and nuclear magnetic resonance (NMR) spectroscopy. Although the HPLC chromatograms indicated that the peptides could easily be separated, it proved difficult to separate the oxidized forms of the peptides from the non-oxidized forms. This is probably due to oxidation occurring in the different purification steps, which was to a degree unavoidable. Such oxidation is a common problem in methionine containing peptides [41]. NMR analysis of the purified peptide fractions revealed that turgencin A_Mox1_ contained 28% turgencin A_Mox0_, turgencin B contained 29% turgencin B_Mox1_ and 4% turgencin B_Mox2_, turgencin B_Mox1_ contained 10% turgencin B_Mox2_ and 2% turegncin B_Mox0_, and turgencin B_Mox2_ contained 3% turgencin B_Mox1_.Turgencin A was not isolated in sufficient amounts for purity analysis using NMR. MS/MS data analyzed over time of the purified peptide showed small changes of the different oxidation states (data not shown). Despite this temporal variation, samples were purified in sufficient amounts for structural determination and peptide sequencing. The variation of methionine oxidation was greater in turgencin B due to the presence of two methionine residues in its sequence, resulting in potentially two possible peptide variants having one Met-ox residue. Tandem MS analysis of turgencin B_Mox1_ indicates that the first methionine is preferentially oxidized, with an absence of a b5 ion for the GIKEM ion at *m/z* 559.29, and a significant signal at *m/z* 575.28 corresponding to the b5 for GIKEM_o_ (Figure S3 in the SI). This preference for oxidation at position 5 is likely due to steric effects as the position of the methionine in position 9 was revealed by NMR to be protected within the peptide structure, compared to the more exposed methionine in position 5.

Methionine residues in peptides are highly susceptible to oxidation even by mild oxidants [42]. This causes a change in the polarity of the amino acid residue, from non-polar to polar, a shift expected to have profound structural and functional consequences [43]. This reaction is known to take place in vivo and to occur during the process of sample preparation and peptide isolation [44]. Whether oxidation of the methionines of turgencin A and B is actively catalyzed by the enzymatic machinery of *S. turgens* is unknown. However, as the herein reported peptides were dissolved in water as part of the isolation process and exposure to air during the drying processes, it is hypothesized that the majority of the peptide fractions with oxidized methionine residues are formed as a result of sample handling. Turgencin A_Mox1_, turgencin B_Mox1_, and turgencin B_Mox2_ are thus believed to be oxidized artefacts of their corresponding unoxidized parent peptides (turgencin A and turgencin B).

### 2.3. Structure Determination

Two-dimensional NMR data was collected for turgencin A_Mox1,_,turgencin B, turgencin B_Mox1_, and turgencin B_Mox2_ in 90:10 H_2_O/D_2_O.

Turgencin A_Mox1_ forms a folded structure in water, resulting in a dispersed ^15^N-HSQC spectrum (Figure S5 in the SI). The resonance dispersion is however less pronounced than for turgencin B (compare Figure S6 in the SI), which appears to have a significant amount of random coil character. The peptide sequence is also significantly less varied than the sequence of turgencin B. For example, seven out of 36 amino acids are lysine. This results in increased spectral overlap, making structural correlations more difficult to deduce unambiguously. The chemical shift assignments were successfully assigned using ^15^N-^1^H and ^13^C-^1^H HSQC, HMBC, TOCSY and HSQC-TOCSY spectra. The sequential assignment was completed through NOE-hopping supported by high-resolution HMBC of the carbonyl region in the carbon dimension where possible (Tables S3 and S4 in the SI). Based on the chemical shift assignments, the protein backbone dihedral angle prediction program TALOS+ was used to predict the secondary structure, resulting in all residues being predicted to be in an unfolded conformation. A quick assessment of the assignable NOE correlations revealed that there were very few structurally important long-range contacts in turgencin A_Mox1_ to base a structure calculation on. For these reasons a full structure determination was not further pursued.

**Figure 3:**
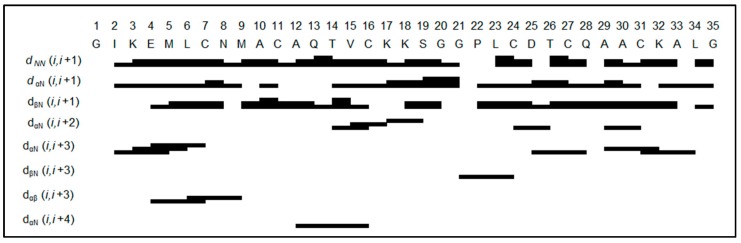
Summary of the sequential NOESY correlations of turgencin B_Mox2_. A total of 69 through-space correlations between neighboring, or near-neighboring, residues were extracted from NOESY experiments using 100, 200, and 300 ms mixing times. The line width in the figure corresponds to the strength of the correlation: the thicker the line, the stronger the NOE. For ‘i, i+2’ and longer range correlations, the lines indicate residue pairs displaying inter-residue correlations.

All turgencin B fractions produced near-identical, well-dispersed natural abundance ^15^N-HSQCs, indicating that they share largely common folded structures that are not substantially affected by the varying numbers of methionine sulfoxides. The turgencin B_Mox2_ data set was the purest fraction of the turgencin B samples and was therefore selected for further structural studies (Figure S6 in the SI). The peptide backbone and side chains were successfully assigned using the same procedure as described above for turgencin A_Mox1_ (Tables S5 and S6 in the SI). The sequential NOESY correlations are summarized in Figure 3.

In total, 108 sequential and 84 long range NOEs were assigned, integrated and classified as strong, medium or weak, applying upper-limit constraints of 2.5, 3.7 and 5.0 Å respectively. Out of these, 16 directly involved alpha- or beta protons of cysteines, which are of extra importance for determining the cysteine connectivity of the peptide. The NOE patterns with relatively strong NN(i,i+1) correlations compared to αN(i,i+1) suggest some helical character, especially when αN(i,i+3) is also detectable. However, there is no long stretch of pronounced alpha helix indicated from the NOE pattern, and, in terms of absolute intensity of the NN(i,i+1) correlations relative to the reference correlations, the helix secondary structure is populated but not a stable fold of the peptide.

TALOS+ was used to predict the secondary structure from the secondary chemical shifts relative to the random coil shifts, using H, NH, CA, CB and CO as input, resulting in a predicted loop-type fold for all residues (Figure S7 in the SI). It should be noted here that the cysteines are relatively evenly distributed in the sequence, and being oxidized their chemical shifts will be different from reduced cysteines. The influence of the disulfide bonds on the quality of the TALOS+ predictions, which is based on the secondary chemical shifts, is unknown to us. The absence of stretches of clear secondary structure was supported by the majority of residues that could be measured [45,46] with ^3^*J*_HNHa_ between 6.0 and 7.5 Hz, which is an indication of conformational averaging between ~4 Hz couplings from θ of -60° (alpha helix) and ~9 Hz from θ in (−120)–(−140)° (beta sheet) (Figure S8 in the SI). All couplings belonging to the stretch of residues between 5 and 12 could only be measured with the TOCSY line width method [45] owing to the structural heterogeneity from the methionine oxidation isomers in combination with the anti-phase pattern of the DQF/E.COSY cross-peaks resulting in severe cancellation. For these reasons, phi and psi dihedral angles were not restrained in the structural calculations, and NOE contacts were treated conservatively with loose restraints to allow conformational flexibility.

**Figure 4:**
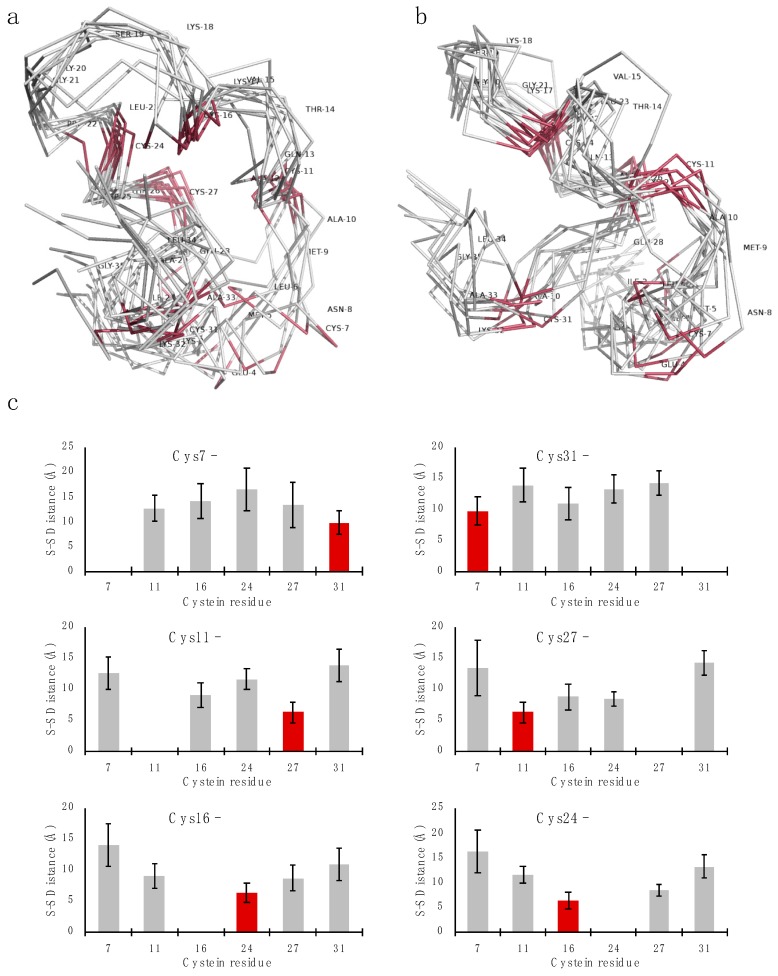
Lowest-energy structure ensembles and average inter-cysteine distances for turgencin B_Mox2_. Panels (**a**) and (**b**) show two views of the 10 lowest energy structure ensembles from simulated annealing with reduced disulfide bonds. Panel (**c**) plots illustrate the average distances from each cysteine sulfur to every other cysteine sulfur, indicating a C1-C6/C2-C5/C3-C4 disulfide pattern, as highlighted with red bars.

To establish the disulfide connectivity pattern by NMR, a preliminary 3D structure was calculated without defining any disulfide bonds. Three iterations of simulated annealing and constraint refinement were performed using an extended starting structure with all cysteines in the reduced form. Out of 100 calculated structures, the 10 lowest energy structures were analyzed for inter-cysteine distances. The ensemble is shown in Figure 4a,b. In the ensemble, the average distances from each cysteine sulfur to every other cysteine sulfur was extracted, indicating a C1-C6/C2-C5/C3-C4 disulfide pattern (Figure 4c).

The disulfide connectivity determined as above was introduced into the structure refinement and the most important stereospecific methyl assignments (valine and leucine) were resolved using the computed structures iteratively. Owing to the intrinsic heterogeneity of the data due to the epimeric oxidized methionines (resulting in four configurational isomers from the combined M5 and M9 chiralities) together with the conformational dynamics, further structural refinement was not pursued past this point. A production run of 500 structures of the main disulfide pattern was calculated using the same simulated annealing protocol, using the best structure with reduced cysteines as starting structure. Several other disulfide connectivity patterns were evaluated in test calculations in terms of energies and evaluated in Figure S9 in the SI. As a more extensive control calculation, 500 structures of the most viable alternative pattern were also calculated using the final production constraints, resulting in overall less-favorable energies (Figure S10 in the SI).

**Figure 5:**
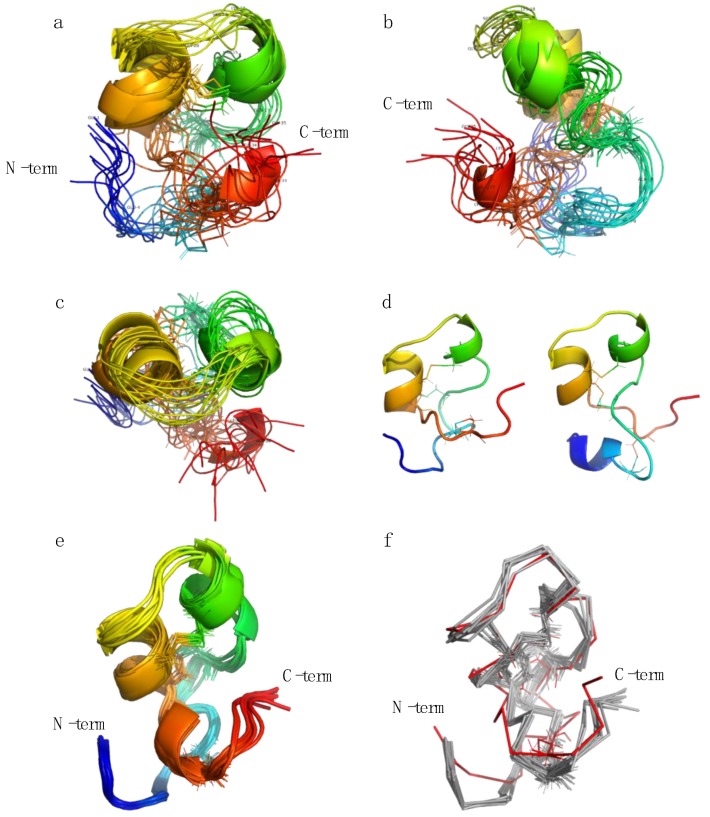
The 10 lowest energy structure ensembles of the major turgencin B_Mox2_ conformers produced by the simulated annealing protocol using the C1-C6/C2-C5/C3-C4 disulfide pattern. Panel (**a**) illustrates the front view, (**b**) side view and (**c**) top view. In panel (**d**) the major conformation (left) is aligned with the minor conformation (right) to illustrate the difference between terminal folds. In the displayed scene the C-terminus folds on top of the N-terminus in the major conformation as opposed to the minor conformation in which the C-terminus is behind the N-terminus. Panel (**e**) shows one representative low energy nuclear magnetic resonance (NMR) structure subjected to 10 ns free molecular dynamics in explicit water and panel (**f**) illustrates a comparison between the NMR structure and the molecular dynamics (MD) structures: in both cases extracted from the final nanosecond of the simulation.

Evaluation of the lowest energy structures revealed two major clusters of conformations, each having distinct folds at their termini and also affecting the outer disulfide bond. The major conformation (~75%) resides in a counter-clockwise twist while the minor conformation (~25%) is in a clockwise twist. Energetically there is no significant difference between the clusters. The 10 lowest energy structures of the major conformation are presented in Figure 5. A comparison overview of the two conformations is presented in Figure 5d.

Structural analysis of the fold reveals that even without constraining any backbone dihedrals, there is a clear tendency to adopt an alpha helix fold for residues 15-18 and 23-27, driven by the NOE constraints alone. These residues do indeed have stronger NOE cross peaks from NH(n) to NH(n-1) than NH(n) to HA(n-1), which is characteristic for alpha helix secondary structure motifs. In many calculated structures there is a helix-like character of various parts of the N-terminal stretch from residue 6-14, and of the C-terminal stretch from 27-31. We interpret this as being a helix fold that is accessible and populated by the peptide in water, but also one that is unstable. Speculatively, the helix fold may very well be an active form that is stabilized by interactions with lipid membranes or other co-factors present in its native environment.

To ensure that the NMR structure was not overly constrained into an unfavorable conformation by the experimental constraints, one representative low energy structure was subjected to an unconstrained 10 ns molecular dynamics (MD) simulation in explicit water, monitoring the energies, conformation and fluctuations. The structure was not significantly different from its starting point and the core residues (defined as residues 6-32, thus ignoring the free termini) stabilized at approximately 1.5 Å backbone rmsd compared to the starting structure (Figure 5e–f and Figures S11 and S12 in the SI). It is noteworthy that the free simulations reinforced the helical character of the peptide rather than unfolding the NMR structure.

To further support the disulfide connectivity of turgencin B as determined by NMR, we attempted to solve it through partial reduction and alkylation, followed by analysis with LC-MS/MS. This however proved to be challenging as the herein used protocol rendered the peptide intact or with all disulfide bridges reduced regardless of incubation conditions evaluated, including varying temperature and length of exposure to the reducing agent.

The disulfide connectivity of the turgencins (C1-C6/C2-C5/C3-C4) is different from the well-characterized human α-defensins (C1-C6/C2-C4/C3-C5) and β-defensins (C1-C5/C2-C4/C3-C6) [47]. However, the overall structural motif of a C1-C6/C2-C5/C3-C4 disulfide linked peptide forming a helix-loop-helix-like structure has been previously observed in peptides from plants, like the viscotoxins from mistletoe [48,49] and crambin from the annual oil seed plant *Crambe abyssinica* [50,51,52]. These, as well as other previously reported C1-C6/C2-C5/C3-C4 peptides, like beta-defensin-like structures from Chinese sea turtles [53,54], typically have two adjacent cysteines involved in separate disulfide bridges and a stabilizing separate structural motif. Turgencin B does however have more evenly distributed cysteines where the C3-C4 pair stabilized the loop and the C2-C5 pair stabilizes the putative helices at the other end. The outermost C1-C6 pair does not have any obvious structural role other than stabilizing the ends. The role of the outermost disulfide bridge, as well as a more stable fold, might be revealed when the peptide interacts with a lipid bilayer or a co-factor. Structurally, turgencin B is not closely related to previously published peptides with the same cysteine patterns (listed in Table S7), but there are some overarching similarities, primarily to the viscotoxins, which are lysine-rich (and hence positively charged) and share the helix-loop-helix motif. However, the evolutionary relationship between the turgencins and other AMPs can only be fully revealed after characterization of their genes. Unfortunately, our attempts to construct a cDNA library have not yet been successful, probably due to degraded RNA (access only to frozen material).

### 2.4. Biological Activity

All isolated peptides showed antibacterial activity but to differing degrees (Table 1), with turgencin A_Mox1_ being the most potent peptide. The minimum inhibitory concentrations (MICs) of turgencin A_Mox1_ against *Corynebacterium glutamicum* and *Bacillus subtilis* were as low as 0.4 µM, and the MIC against *Escherichia coli* was 0.8 µM. In addition, turgencin A_Mox1_ affected eukaryotic cell viability, exhibiting an IC_50_ value against the human melanoma cancer cell line A2058 of 1.4 µM, and an IC_50_ against the non-malignant human fibroblast cell line MRC-5 of 4.8 µM. These results show that turgencin A_Mox1_ is cytotoxic, but the MIC against the most sensitive bacteria is still 12 times lower than the IC_50_ against the non-malignant cell line.

The bioactivities of turgencin B and the two oxidized analogues (turgencin B_Mox1_ and turgencin B_Mox2_) vary depending on the total number and positions of the oxidations. It was found that there was an inverse relationship between the numbers of methionine sulfoxides harbored by the turgencins and their respective bioactivities, a common feature among peptides with methionine oxidation [55]. According to NMR analysis, methionine oxidation did not cause significant changes in the conformation of the peptides. Methionine oxidation changes the polarity of this amino acid residue from non-polar to polar. The observed reduction in bioactivity in the oxidized variants of turgencin B compared to turgencin B_Mox0_ may be caused by a break in the lipophilic section of the peptide, resulting in insufficient affinity for the lipophilic portion of the bacterial and mammalian cell envelope, consequently reducing the antibacterial and cytotoxic effects of the oxidized variants. Despite this inverse relationship, when comparing antimicrobial activity and cell viability it might be argued that turgencin B_Mox1_, with one Met-ox, might be a better drug-lead candidate than the unoxidized turgencin B. Even though the antibacterial effect of turgencin B_Mox1_ is slightly lower than that of turgencin B, turgencin B_Mox1_ did not affect the non-malignant cell line at the highest concentration tested (50 µM). With both methionines oxidized, as in turgencin B_Mox2_, the MICs across all cell lines were considerably higher. The un-oxidized turgencin A might therefore be even more potent than turgencin A_Mox1,_ which has one Met-ox. Unfortunately, only minor amounts of turgencin A were observed during liquid chromatography-mass spectrometry (LC-MS) analysis (Figure S2), and the peptide could therefore not be isolated in sufficient quantities to allow for assessment of its bioactivities. These changes in bioactivity indicate that peptides containing methionine may be poor drug candidates. Niederhafner et al. [55] replaced methionine with norleucine in the peptide melectin and improved its antimicrobial activity, but it also increased the cytotoxic activity towards red blood cells from rats. Thus, although it is outside the scope of the current study, future work should investigate whether analogues without methionines exhibit improved bioactivity profiles.

### 2.5. Real-time Measurement of Immediate Effect on Membrane Integrity and Viability

**Figure 6:**
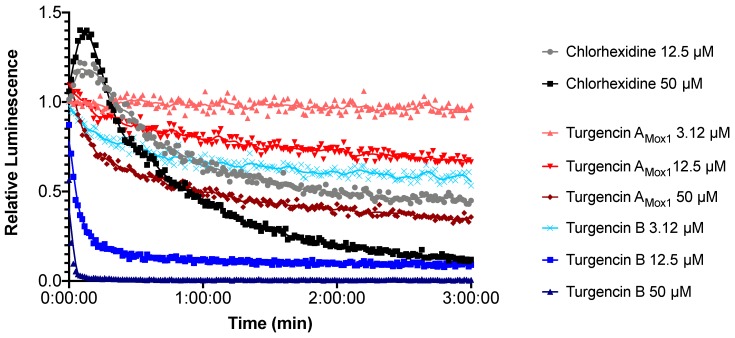
Kinetics of the antimicrobial effect on membrane integrity as measured by relative luminescence emission in *B. subtilis* (pCSS962). Cells were treated with turgencin A_Mox1_(shades of red), turgencin B (shades of blue) or chlorhexidine. Each data point is the mean of three independent measurements normalized to the water negative control. Chlorhexidine was used as positive control and its addition in the assay elicited an initial peak in luminescence followed by a rapid drop below the background typical of membrane active substances. Turgencin A_Mox1_ reduced light emission at concentrations above 12.5 µM while turgencin B reduces light emission more efficiently and at concentrations of 3.12 µM and above. Note that the starting point depicts the start of luminescence measurement, not the combination of cells and analyte, which occurs approximately 10 s prior.

Bacterial biosensors (*B. subtilis* and *E. coli*, both carrying the pCSS962 plasmid with the LucGR gene) were used to assess the immediate membrane disruptive properties of turgencin A_Mox1_ and turgencin B. The strains express eukaryotic luciferase and will emit a luminescence peak if their membranes are disrupted and externally added D-luciferin is allowed to diffuse into the cell [56]. The luminescence measurements of *B. subtilis* after exposure to turgencins or chlorhexidine are shown in Figure 6. Both peptides affected the bacterial membrane integrity of *B. subtilis*. Although no increase in light emission was observed, except for chlorhexidine, a decrease in light intensity was observed for both peptides. Exposure to the natural peptide turgencin A_Mox1_ (Figure 6) decreased luminescence by ~50% after 1 minute at a concentration of 50 μM (corresponding to 125 × MIC). Turgencin B exposure reduced light emission even more profoundly and much faster. Within 10 s of the addition of the peptide at a concentration of 50 μM, bacterial luminescence was reduced by 97%. Additionally, the effect was clearly dose dependent. At concentrations above 6.25 μM (4 × MIC) turgencin B reduced relative luminescence by more than 50% within 1 minute (Figure 6). For fast-acting membrane active substances, it was not possible to resolve the initial peak in light emission as the lag time between addition of the cells to the analyte and first plate reading is approximately 10 s due to constraints of the plate reader. Therefore, only the subsequent drop below background luminescence could be observed. Our results indicate that addition of the peptide elicits a rapid effect on cell viability. None of the peptides displayed any pronounced effect on the membrane of *E. coli*; only a weak effect of turgencin B at 50 μM (4 × MIC) concentration was observed (data not shown). Chlorhexidine, an antiseptic agent known for its membrane-disruptive properties [57], produced an immediate increase in luminescence (caused by an increased influx of D-luciferin into the cells) and subsequently a decrease at concentrations of 12.5 μM and above (Figure 6).

In order to independently confirm the bactericidal effect observed in the membrane integrity assays, real-time cell viability assay was performed using bacterial biosensors carrying either a chromosomally integrated (*B. subtilis* 168) or a plasmid-borne (*E. coli* K12) *lux* operon. Both strains express a bacterial luciferase and fatty acid reductases for regeneration of long-chain fatty aldehydes, which serve as substrates for light production. Light production is therefore linked to several metabolic processes, which in turn depend on the regeneration of reduction equivalents and adenosine triphosphate (ATP) [58,59]. While light production indicates active metabolism, loss of light production indicates a decrease in metabolic activity, and hence, reduced viability of the cells.

**Figure 7:**
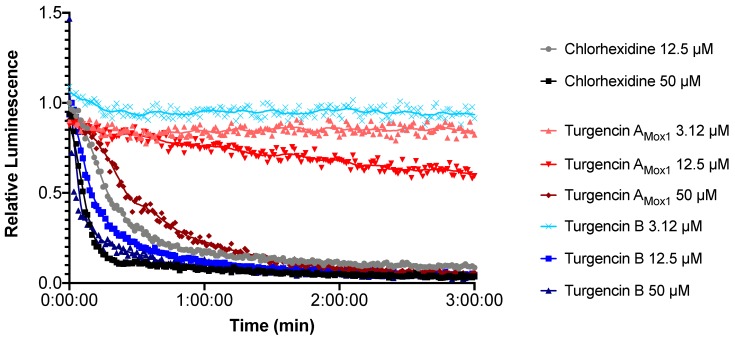
Kinetics of antimicrobial effect on viability of *B. subtilis* as relative luminescence emission from *luxABCDE* treated with turgencin A_Mox1_ (shades of red) and turgencin B (shades of blue). Each data point is the mean of three independent measurements normalized to the water negative control. Chlorhexidine and water were used as positive and negative controls, respectively.

**Figure 8:**
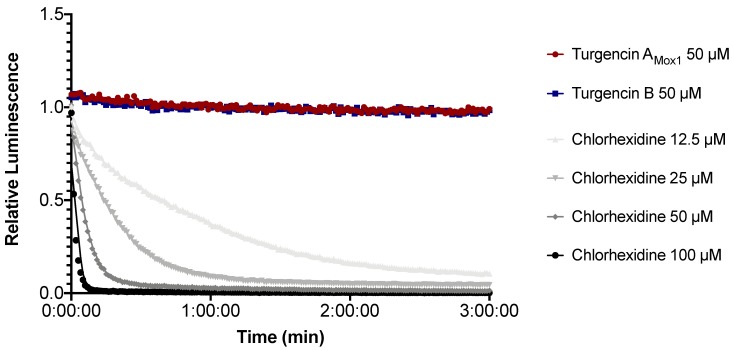
Kinetics of the relative luminescence emission by *E. coli* expressing a *luxCDABE* operon (pCGLS-11) treated with turgencin A_Mox1_ or turgencin B. Each data point is the mean of three independent measurements. Chlorhexidine and water were used as positive and negative controls, respectively.

The immediate effect of turgencins on the viability of *B. subtilis* is presented in Figure 7. The results show that they both affect the viability of the Gram-positive strain within the assay period. Turgencin A_Mox1_ and turgencin B caused more than a 50% decrease in light production after 3 min of incubation at concentrations above 25 µM (12.5 × MIC) and 6.25 µM (4 × MIC), respectively. Furthermore, the observed effect was dose-dependent, with decreased light production observed at higher concentrations. Chlorhexidine treatment caused >50% reduction in light production at concentrations of 6.25 µM and above (10 × MIC, unpublished results). The decrease in light emission within 3 min at concentrations above the MIC confirms that cell viability is affected relatively fast. The same experiment conducted in *E. coli* showed that, in contrast to chlorhexidine, the turgencin peptides did not affect the viability of this strain within the 3 min assay period at 50 µM concentrations (Figure 8).

## 3. Materials and Methods 

### 3.1. Materials

Live specimens of the sea squirt *Synoicum turgens* (Phipps, 1774) were collected off the coast of Svalbard in October 2011 (79°31′N, 18°45′E) and August 2016 (79°33′N, 18°37′E) by divers at 20–30 m depth, and outside of Bjørnøya in May 2009 (74°28′N, 18°44′E) by divers at 20 m depth and by Agassiz trawl (74°20′N, 19°20′E) at 47 m depth. All four samples were identified by Robert A. Johansen at the Norwegian national biobank (Marbank), frozen separately at −20 °C at sea, lyophilized, and again frozen until extraction. Voucher specimens (reference numbers: M11HEL0441, M16HEL1403, M09JAN0062-4 and M09JAN0059) were deposited in Marbank, Tromsø, Norway.

### 3.2. Extraction of Antimicrobial Peptides

Freeze-dried samples (100 g) were extracted twice with five volumes (v/w) of 60% (v/v) MeCN (HPLC-grade, Sigma-Aldrich, Steinheim, Germany) containing 0.1% trifluoroacetic acid (TFA; Sigma-Aldrich) for 24 h at 4 °C. The combined supernatants were incubated at −20 °C for approximately 1 h allowing two liquid phases, an organic, MeCN-rich phase and an aqueous, salt-rich phase to be formed and separated. The aqueous phase was dried in a ScanSpeed 40 vacuum centrifuge (LabogeneApS, Denmark), and resuspended in 0.05% TFA/H_2_O (v/v) to a concentration of 100 mg/mL. Salt was removed from the aqueous phase by SPE, as previously described [60]. Briefly, the extracts were loaded onto reversed-phase C18 35cc Sep-Pak Vac cartridges (Waters, MA, USA) equilibrated with 0.05% TFA/H_2_O (v/v). After washing with acidified water, the analytes were eluted with 10, 20, 30, 40, and 80% (v/v) MeCN containing 0.05% TFA (v/v). The SPE eluates were dried in a ScanSpeed 40 vacuum centrifuge, resuspended in MQ-H_2_O (Millipore, MA, USA), and tested for antimicrobial activity.

Antimicrobial SPE eluates (the 40 and 80% MeCN eluate) were further fractionated by preparative high-performance liquid chromatography (HPLC) on an Agilent 218 Preparative LC system coupled to an Agilent 1260 infinity DAD with an Agilent 440-LC fraction collector (Matriks, Oslo, Norway). Separation was achieved using a Waters XBridge BEH C18 Prep Column (10 × 250 mm, 5 µm) column. The mobile phase consisted of two eluents: eluent A, H_2_O with 0.1% formic acid (FA, Pro-analysis, Sigma-Aldrich), and eluent B, MeCN with 0.1% FA. The peptides were separated using a linear gradient of 5-60% eluent B over 60 min, with a flow rate of 6 mL/min. Fractions (6 mL) were collected at regular 1 min intervals, which were then dried in a ScanSpeed 40 vacuum centrifuge, resuspended in MQ-H_2_O, and tested for antimicrobial activity.

Active HPLC fractions were submitted to an Agilent 1290 Infinity UHPLC system, coupled to an Agilent high resolution 6540B quadrupole time-of-flight (Q-ToF) mass spectrometer with a dual electrospray ionization (ESI) source, controlled by MassHunter software (Matriks), for identification of the bioactive constituents. The MS analysis revealed semi-purified peptides which required further purification using an analytical HPLC-DAD-MS system consisting of a 600 Pump, a 2996 Photodiode Array UV detector, a 3100 single quadrupole mass detector, and a 2767 sample manager (Waters, MA, USA). The system was controlled by MassLynx 4.1 and FractionLynx application manager. A linear gradient was developed for optimal separation, consisting of 20–36% MeCN (with 0.1% FA) over 24 min and a flow rate of 6 mL/min using a Waters XBridge BEH C18 Prep Column (10 × 250 mm, 5 μm). The resulting ion chromatograms of the peptides were used to determine the retention times (RTs; i.e. relative hydrophobicity) of the bioactive peptides. Fractions containing semi-purified peptides were collected using the “timed-event” function of the MassLynx software. Peptide fractions were pooled, dried in a ScanSpeed 40 vacuum centrifuge, and resuspended in MQ-H_2_O. Purity was checked with analytical HPLC, UHPLC-QToF-MS and NMR, and the antimicrobial activity was profiled.

### 3.3. Peptide Sequencing

Primary structure determination of the peptides turgencin A_Mox1_ and turgencin B_Mox2_ was performed at Eurosequence (Groningen, The Netherlands, www.eurosequence.nl). The complete sequence of turgencin B_Mox2_ was obtained solely by Edman degradation sequencing, whereas the sequence of turgencin A_Mox1_ was obtained by a combination of N-terminal Edman degradation, reduction and alkylation of the peptide with iodacetamide, followed by Glu-C digestion, separation of fragments by RP-HPLC, and finally analyzing the obtained fragments by Edman degradation and MS/MS. The peptide sequences have been submitted to the UniProt Knowledgebase with the accession numbers C0HLN5 (turgencin A) and C0HLN6 (turgencin B).

### 3.4. Partial Reduction and Alkylation

Lyophilized turgencin B (0.5 mg) was partially reduced in 210 µL of 0.17 M citrate buffer (32 mg/mL citric acid, 50 mg/mL trisodium citrate dihydrate) (pH 3.0). Peptide sample was then combined 1:1 with cold (4 °C) 10 mM tris(2-carboxyethyl)phosphine (11.4 mg/mL) solution in 0.17 M citrate buffer (pH 3.0). One sample of turgencin B was incubated at 40 °C, a second sample at room temperature, and a third sample at 4 °C. Aliquots of 50 µL were taken out at different timepoints (10, 20, 30, 45, 60, 90, 120 and 240 min) from all three samples. Upon sampling, each reaction solution was immediately added to 0.6 mg of pre-prepared N-ethylmaleimide (NEM) and incubated for 15 min at 37 °C before acidification through addition of 300 µL of 1% FA. After this step, salt was removed from all samples using C18 ZipTips (Millipore), and eluted in 10 µL of 80% MeCN with 1% FA. Following this, 100 µL of 0.5 M ammonium bicarbonate (pH 8.0) was added. Samples were fully reduced by adding 35 µL of 100 mM dithiothreitol, deoxygenated with N_2_, and incubated at 60 °C for 30 min. Afterwards, they were alkylated by adding 35 µL of 250 mM iodoacetamide for 15 min at room temperature. The reduced and alkylated samples were analyzed on a Nexera UHPLC (Shimadzu) coupled to a TripleToF 5600 MS (AB Sciex).

### 3.5. NMR Spectroscopy and Calculations

All NMR experiments were performed on a Bruker Avance III HD spectrometer equipped with an inverse four-channel probe with cryogenic enhancement for ^1^H, ^2^H and ^13^C (TCI) operating at 600 MHz for ^1^H (Bruker Biospin, Switzerland). When applicable, gradient-selected experiments with adiabatic pulse sequences were used. The experiments acquired for the studied peptides were Presat, Excitation sculpting, Carbon, ^15^N-HSQC, ^13^C-HSQC, HMBC, band-selective HMBC (carbonyl hires), H_2_BC, HSQCTOCSY (60 ms DIPSI), NOESY (100, 200, 300 ms mixing time), ROESY (100 ms spinlock), DQF-COSY, E.COSY and TOCSY (20, 60, 100 ms DIPSI). Acquisition and processing were done in Topspin 3.5pl7 using standard pulse sequences (Bruker Biospin, Switzerland). The NMR samples were prepared by dissolving 1–2 mg of peptide in 120 µL ultra-pure water and adding a drop of D_2_O in a 3 mm Shigemi tube matched for D_2_O. Spectral assignments and integrations were performed in CARA 1.8.4.2. Secondary structure prediction was made in TALOS+.

Structures were generated using XPLOR-NIH 2.52. Starting structures were created as extended chains and folded using standard simulated annealing protocol (2000 K, 20000 cooling steps in vacuo) using NMR-derived constraints without connecting any disulfide bonds. Low energy folds were then used to generate disulfide connected starting structures for the final refinements. Finally, production runs of 500 cycles of simulated annealing was used to generate the reported structure ensemble. Representative low energy structures were then solvated in an orthorhombic water box of explicit SPC water and neutralized by adding 3 Cl- ions, with 10 × 10 × 10 Å clearance from the peptide atoms using the Desmond package in Maestro v11.4.011, MMshare v4.0.011, release 2017-4 for the Schrodinger suite. The structure was minimized (min 10 SD steps/3 LBFGS vectors) before a 10 ns free MD (2 fs timesteps, 9.0 Å electrostatics cutoff) was simulated under constant pressure and temperature (300 K Nose-Hoover thermostat, Martyna-Tobias-Klein barostat). The NMR data of this paper are available at the Biological Magnetic Resonance Data Bank under accession number 50153.

### 3.6. Microbial Strains and Antibacterial Activity Assays

The Gram-negative bacteria *E. coli* (*E.c*, ATCC 25922) and *P. aeruginosa* (*P.a*, ATCC 27853), and the Gram-positive bacteria *S. aureus* (*S.a*, ATCC 9144), *C. glutamicum* (*C.g*, ATCC 13032), and *B. subtilis* (*B.s*, ATCC 23857) were used as test bacteria. Cultures stored in glycerol at −80 °C were transferred to Mueller–Hinton (MH) plates (Difco Laboratories, Detroit, MI, USA) and incubated at room temperature for two days. One colony from each bacterial strain was transferred to 5 mL MH broth and shaken at 600 rpm overnight. From these vials of actively growing bacteria, 20 µL were transferred to 5 mL MH broth and shaken for 2 h at room temperature. The antibacterial assays were performed as previously described [61], but with the following exception; bacterial cultures were diluted with medium to 2.5–3.0 × 10^4^ bacteria/mL concentrations. An aliquot of 50 µL (1250–1500 bacterial cells) was added to each well in 96 microwell plates (Thermo Fisher Scientific, Denmark), preloaded with sample solution (peptides or eluates) and controls (water, medium or antibiotic). The plates were incubated at 35 °C for 24 h, using an EnVisionMultilable Reader, controlled by EnVision Manager (PerkinElmer, United Kingdom) to measure the optical density (OD_595_) every hour. The minimum inhibitory concentration (MIC) was set to the point a sample showed >90% reduction in OD_595_ after 24 h compared to the growth control (bacteria plus MQ-H_2_O). Oxytetracycline (Sigma-Aldrich, Steinheim, Germany) in a serial dilution was used as a positive (antibacterial) control (40-0.04 µM). All experiments were performed in triplicates. The highest peptide concentration tested against the bacteria was 100 µM, and the lowest was 0.02 µM.

### 3.7. Real-time Assay Measuring Immediate Membrane Disruption

A real-time membrane integrity assay (modified from [56]) was performed using *B. subtilis* 168 (ATCC 23857) and *E. coli* K12 (ATCC MC1061), both carrying the plasmid pCSS962 with the eukaryotic luciferase gene *lucGR*. The luciferase enzyme is dependent on D-luciferin as substrate to emit light. Externally added D-luciferin (MW 280 Da) does not penetrate intact cell membranes efficiently. However, disruption of the bacterial membrane integrity (like pore-formation) will lead to the influx of luciferin and subsequently induce bacterial light emission. After reaching a peak, the light intensity will reduce and drop below normal values (intact cell membranes) due to reduced cell numbers.

*B. subtilis* and *E. coli* were cultured overnight in MH broth supplemented with 5 µg/mL chloramphenicol (Merck KGaA, Darmstadt, Germany) and a mixture of 20 μg/mL chloramphenicol / 100 μg/mL ampicillin, respectively, before being centrifuged at 4500 rpm for 10 min. The supernatant was removed, and the pellet resuspended in MH broth to give an OD_600_ of 0.1. D-luciferin potassium salt (Synchem Inc., Elk Grove Village, IL, USA) at a final concentration of 1 mM was added to the bacterial cultures and the background luminescence was measured. Twofold dilutions (final assay concentration of 50–1.6 µM) of turgencin A_Mox1_ and B dissolved in MQ-H_2_O were prepared and added (10 µL) to black round-bottom 96-well microtiter plates (Nunc, Roskilde, Denmark). Chlorhexidine acetate (Fresenius Kabi, Halden, Norway), at assay concentrations of 200–12.5 µM, was used as a positive control, whereas MQ-H_2_O was used as a negative control. The plates were loaded into a Synergy H1 Hybrid Reader (BioTek, Winooski, VT, USA). Aliquots (90 µL, to give a total assay volume of 100 µL) of the prepared bacteria suspension were added to the test wells by an automatic injector and luminescence emission was recorded every second for 180 s. All experiments were performed in triplicate and all values were normalized to untreated water controls.

### 3.8. Real-time Assay Measuring Bacterial Cell Viability

A real-time cell viability assay (modified from [62]) was performed using *B. subtilis* 168 and *E. coli* K12, carrying a chromosomal integration of an optimized *lux*ABCDE operon controlled by the constitutive promoter P*veg* [63] or the plasmid pCGLS-11 [64] with the *luxCDABE*operon from *Xenorhabdus luminescens*, respectively. These strains express luciferase constitutively and emit light as long as they are alive (i.e. have a functioning metabolism). Thus, adding a bactericidal compound will result in reduced light emission due to reduced cell viability and/or cell number. *B subtilis* and *E. coli* were cultured overnight in MH broth supplemented with 5 µg/mL chloramphenicol and 100 μg/mL ampicillin, respectively, before being centrifuged at 4500 rpm for 10 min. The supernatant was removed, and the pellet resuspended in MH broth to give an OD_600_ of 0.1. Twofold dilutions (10 µL) of turgencin A_Mox1_ and turgencin B (assay concentrations: 50–1.6 µM) and the positive control chlorhexidine acetate (assay concentrations: 200–12.5 µM) were added to black round-bottom 96-well microtiter plates (Nunc). MQ-H_2_O was used as a negative control. The plates were loaded into the Synergy H1 Hybrid Reader and aliquots (90 µL, to give a total assay volume of 100 µL) of the bacteria suspension were added to the test wells by an automatic injector. Luminescence emission was subsequently measured every second for 180 s. All experiments were performed in triplicate. The measurements were normalized to the untreated water controls.

### 3.9. Human Cell Viability Assay

The human melanoma cancer cell line A2058 (ATCC CRL-11147TM) and the non-malignant human fibroblast cell line MRC-5 (ATCC CCL-171) were cultured in Roswell Park Memorial Institute-1640 (RPMI; VWR, West Chester, PA, USA) medium, supplemented with 10% heat-inactivated fetal bovine serum (FBS; VWR, West Chester, PA, USA) at 37 °C in a humidified atmosphere of 5% CO_2_. The inhibitory effect of the peptides on the proliferation on A2058 and MRC-5 were assessed. Cells were plated in 96-well microtiter plates at an initial density of 2000 (A2058) or 4000 (MRC-5) cells per well and allowed an overnight period for attachment. Cell media were then removed and replaced with fresh RPMI media containing ranging concentrations of the peptides. Cells were then incubated for 72 h at 37 °C. To each well 10 µL AqueousOne (Promega, Madison, WI, USA) was added 1 h before the end of the incubation period. Absorbance was measured at 485 nm. Cells in compound-free RPMI medium were used as a negative control and cells treated with 0.1% Triton® X-100 reagent (Sigma Aldrich, Steinheim, Germany) were used as a positive control. All experiments were performed in triplicate and repeated three times.

## 4. Conclusions

We report the isolation, structural and biochemical characterization of several novel cysteine rich AMPs from *S. turgens*. From a structural perspective, neither of the peptides, turgencin A and turgencin B, was found to exhibit sequence homology to any previously reported peptides, even with the same disulfide connectivity, however they do display overarching similarities to the lysine-rich viscotoxins which are also positively charged and share the helix-loop-helix motif. Antimicrobial activities and cytotoxicities across various oxiforms of these new peptides varied considerably, and hence future studies should elaborate and examine the importance of the methionine residues for the bioactivities of turgencins. To our knowledge, the turgencins are the first cysteine-rich AMPs ever isolated and characterized from ascidians/tunicates.

## Figures and Tables

**Table 1:** Antimicrobial and cytotoxic activity of the isolated turgencins.

Peptide	Antimicrobial Activity (MIC; µM)					Cytotoxic Activity (IC_50_; μM)	
	*C. g.*	*B. s.*	*S. a.*	*E. c.*	*P. a.*	A2058	MRC-5
Turgencin A_Mox1_ (72% Mox1, 28% Mox0)	0.4	0.4	6.3	0.8	1.6	1.4	4.8
Turgencin B (67% Mox0, 29% Mox1, 4% Mox2)	1.6	1.6	>100.0	12.5	25.0	4.1	7.5
Turgencin B_Mox1_ (88% Mox1, 10% Mox2, 2% Mox0)	1.6	3.1	>100.0	25.0	>100.0	27.4	>50.0
Turgencin B_Mox2_ (97% Mox2, 3% Mox1)	25.0	25.0	>100.0	>100.0	>100.0	>50.0	>50.0

*C.g.—Corynebacterium glutamicum, B.s.—Bacillus subtilis, S.a.—Staphylococcus aureus, E.c.—Escherichia coli, P.a.—Pseudomonas aeruginosa*, A2058—Human melanoma cancer cell line and MRC-5—Non-malignant human fibroblast cell line.

## Supplementary Materials

The following are available online at https://www.mdpi.com/1660-3397/18/1/51/s1, Figure S1: MS spectra of the isotope patterns of the purified peptides, Figure S2: MS/MS spectra of the intact peptides of turgencin A and turgencin A_Mox1_, Figure S3: MS/MS spectrum highlighting the different oxidation states of turgencin B, Figure S4: Retention times of turgencin A, B and their oxidized derivatives, Figure S5: ^15^N-HSQC (at natural abundance) of turgencin A_Mox1_ in water, Figure S6: ^15^N-HSQC (at natural abundance) of turgencin B_Mox2_ in water, Figure S7: TALOS+ predicted secondary structure of turgencin B_Mox2_, Figure S8: The ^3^*J*_HNHA_ coupling constants of turgencin B_Mox2_, Figure S9: Early test calculations of turgencin B_Mox2_ using crude constraints to evaluate different disulfide patterns using crude constraints, Figure S10: The most viable alternative disulfide pattern of turgencin B_Mox2_ compared to the found pattern in terms of energy, Figure S11: RMSD relative to the starting frame, energies of the turgencin B_Mox2_ and the RMSF of the backbone during the free MD trajectory, Figure S12: Comparison of NMR and molecular dynamics structures for turgencin B_Mox2_, Table S1: Antimicrobial activity of solid phase extract fractions and the organic extract, Table S2: Calculated and measured monoisotopic *m/z* [M+4H]^4+^ ions of the turgencins, Table S3: Proton chemical shift assignments for turgencin A_Mox1_ in water, Table S4: Carbon chemical shift assignments for turgencin A_Mox1_ in water, Table S5: Proton chemical shift assignments for turgencin B_Mox2_ in water, Table S6: Carbon chemical shift assignments for turgencin B_Mox2_ in water, Table S7: Peptides sharing the same disulfide connectivity as turgencin B.
