# Supplementary material for: Isolation and Characterization of Antimicrobial Peptides with Unusual Disulfide Connectivity from the Colonial Ascidian Synoicum turgens
Source: Mar Drugs. 2020 Jan 12;18(1):51. doi: 10.3390/md18010051 (PMC7024374; doi:10.3390/md18010051)
Supplement: Supplementary file 1 [file marinedrugs-18-00051-s001.pdf]

Supporting information

Isolation and characterization of antimicrobial peptides with unusual disulfide connectivity from the colonial ascidian *Synoicum turgens*

Ida K. Ø. Hansen [1], Johan Isaksson [2], Aaron G. Poth[3], Kine Ø. Hansen [4], Aaron J. C. Andersen [1], Céline S. Richard [1], Hans-Matti Blencke[1], Klara Stensvåg[1], David J. Craik[3] and Tor Haug [1]

[1] Norwegian College of Fishery Science, Faculty of Biosciences, Fisheries and Economics, UiT The Arctic University of Norway, Breivika, N-9037 Tromsø, Norway
[2] Department of Chemistry, UiT The Arctic University of Norway, Breivika, N-9037 Tromsø, Norway
[3] Institute for Molecular Bioscience, The University of Queensland, Brisbane 4072, Queensland, Australia
[4] Marbio, UiT The Arctic University of Norway, Breivika, N-9037, Tromsø, Norway

Table of contents

Figure S1. MS spectra of the isotope patterns of the purified peptides
Figure S2. MS/MS spectra of the intact peptides of turgencin A and turgencin A_{Mox1}
Figure S3. MS/MS spectrum highlighting the different oxidation states of turgencin B
Figure S4. Retention times of turgencin A, B and their oxidized derivatives
Figure S5. ^{15}N-HSQC (at natural abundance) of turgencin A_{Mox1} in water
Figure S6. ^{15}N-HSQC (at natural abundance) of turgencin B_{Mox2} in water
Figure S7. TALOS+ predicted secondary structure of turgencin B_{Mox2}
Figure S8. The $^3J_{HNHA}$ coupling constants of turgencin B_{Mox2}
Figure S9. Early test calculations of turgencin B_{Mox2} using crude constraints to evaluate different disulfide patterns using crude constraints
Figure S10. The most viable alternative disulfide pattern of turgencin B_{Mox2} compared to the found pattern in terms of energy
Figure S11. RMSD relative to the starting frame, energies of the turgencin B_{Mox2} and the RMSF of the backbone during the free MD trajectory
Figure S12. Comparison of NMR and molecular dynamics structures for turgencin B_{Mox2}
Table S1. Antimicrobial activity of solid phase extract fractions and the organic extract
Table S2. Calculated and measured monoisotopic *m/z* $[M+4H]^{4+}$ ions of the turgencins
Table S3. Proton chemical shift assignments for turgencin A_{Mox1} in water
Table S4. Carbon chemical shift assignments for turgencin A_{Mox1} in water
Table S5. Proton chemical shift assignments for turgencin B_{Mox2} in water
Table S6. Carbon chemical shift assignments for turgencin B_{Mox2} in water
Table S7. Peptides sharing the same disulfide connectivity as turgencin B

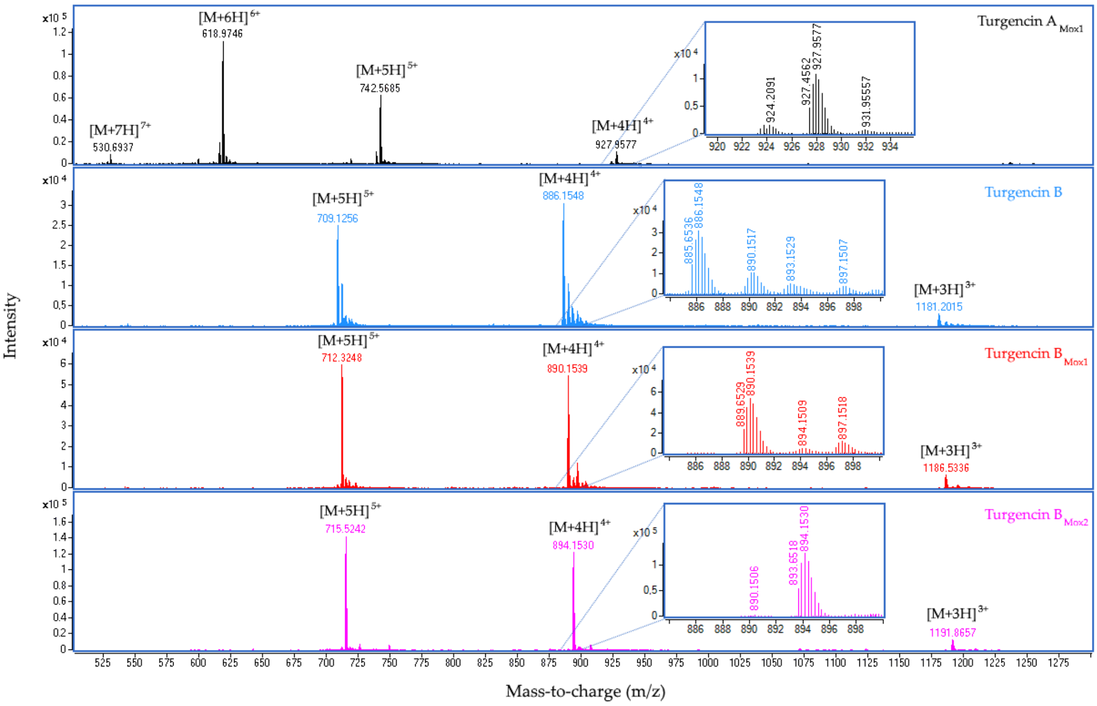

Figure S1. MS spectra of the isotope patterns of the purified peptides turgencin A$_{Mox1}$, turgencin B, turgencin B$_{Mox1}$ and turgencin B$_{Mox2}$. The [M+4H]$^{4+}$ ions are highlighted, and the monoisotopic signals of these ions were used for calculation of the monoisotopic masses of the peptides.

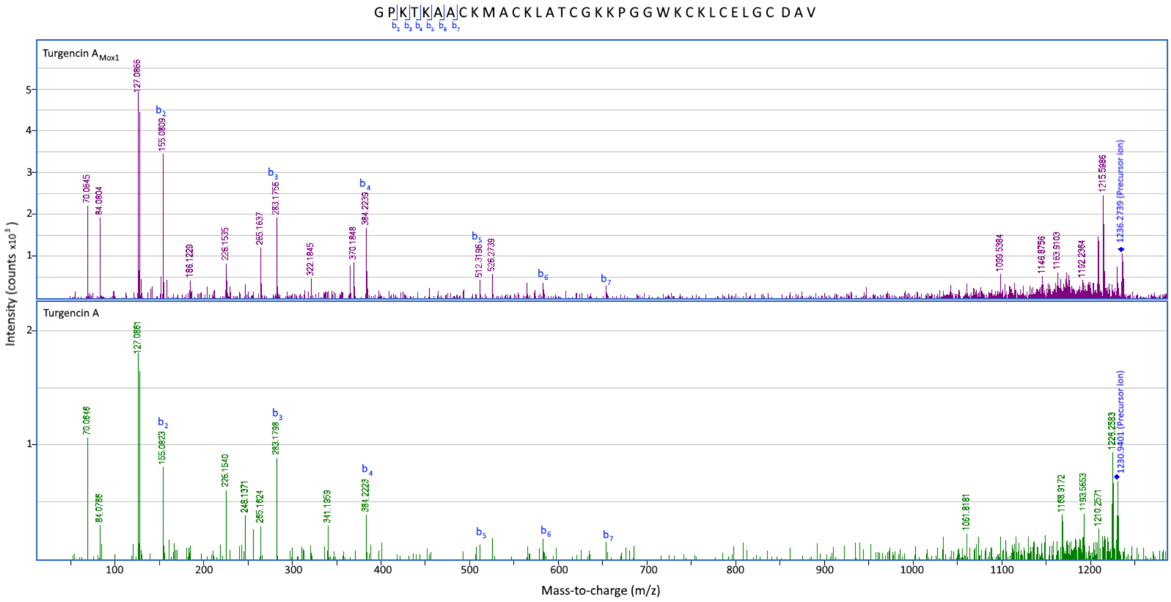

Figure S2. MS/MS spectra of the [M+3H]$^{3+}$ precursor ions *m/z* 1236.27 and *m/z* 1230.94 of the intact peptides of turgencin A and turgencin A$_{Mox1}$ respectively.

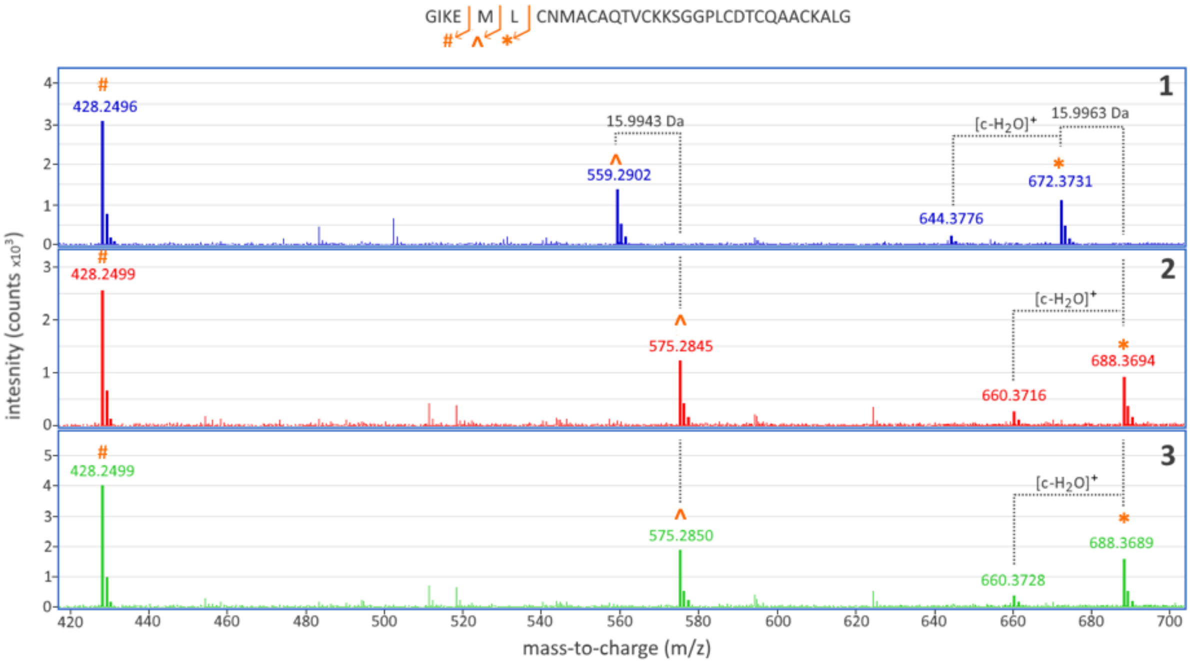

Figure S3. MS/MS spectra of the $[M+4H]^{4+}$ precursor ions, highlighting the differing oxidation states of Met5 across three turgencin B oxiforms, zoomed in between *m/z* 420-700. Spectrum 1 is of turgencin B (precursor *m/z* 885.65), spectrum 2 illustrates the oxidation of turgencin B_{Mox1} (precursor *m/z* 889.65), and spectrum 3 is of turgencin B_{Mox2} (precursor *m/z* 893.65).

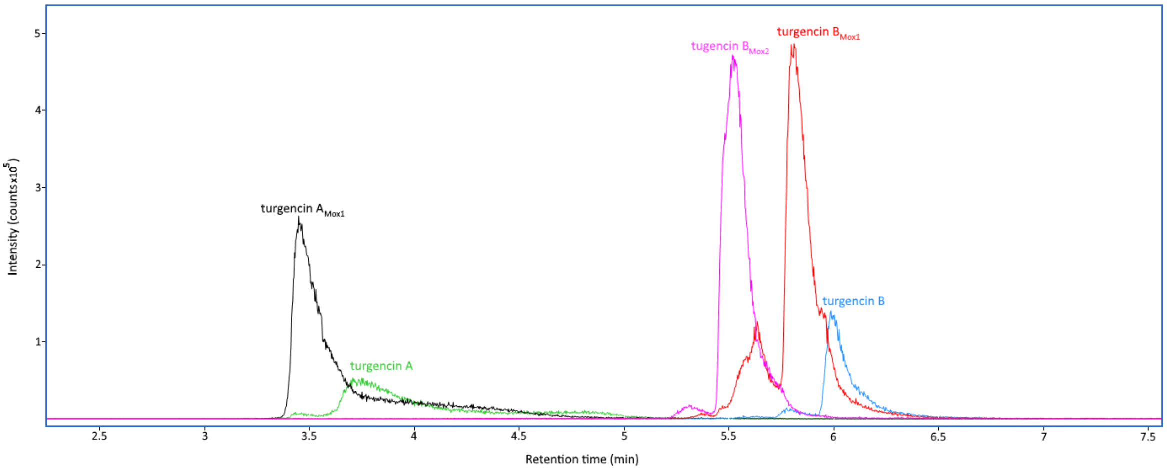

Figure S4. Reversed-phase HPLC separation of turgencin A, B and their oxidized derivatives. Peptides with methionine oxidation exhibit shorter retention times, corresponding with decreased hydrophobicity.

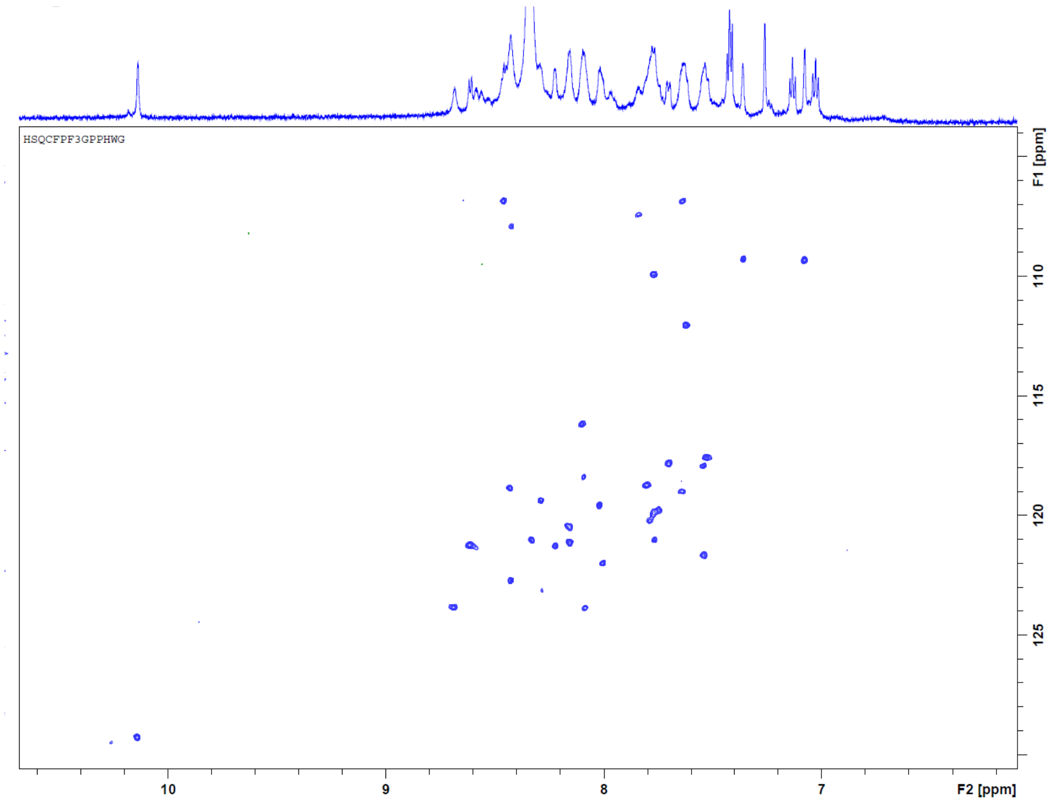

Figure S5. ^{15}N-HSQC (at natural abundance) of turgencin A_{Mox1} in water.

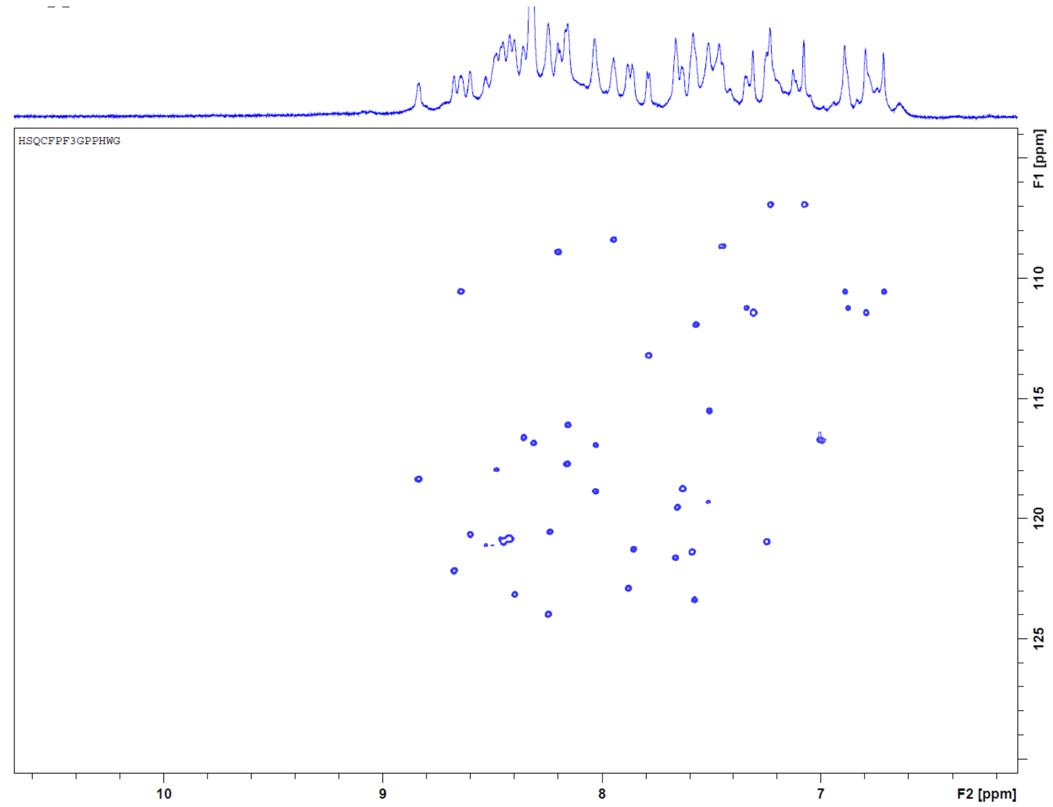

Figure S6. ^{15}N-HSQC (at natural abundance) of turgencin B_{Mox2} in water.

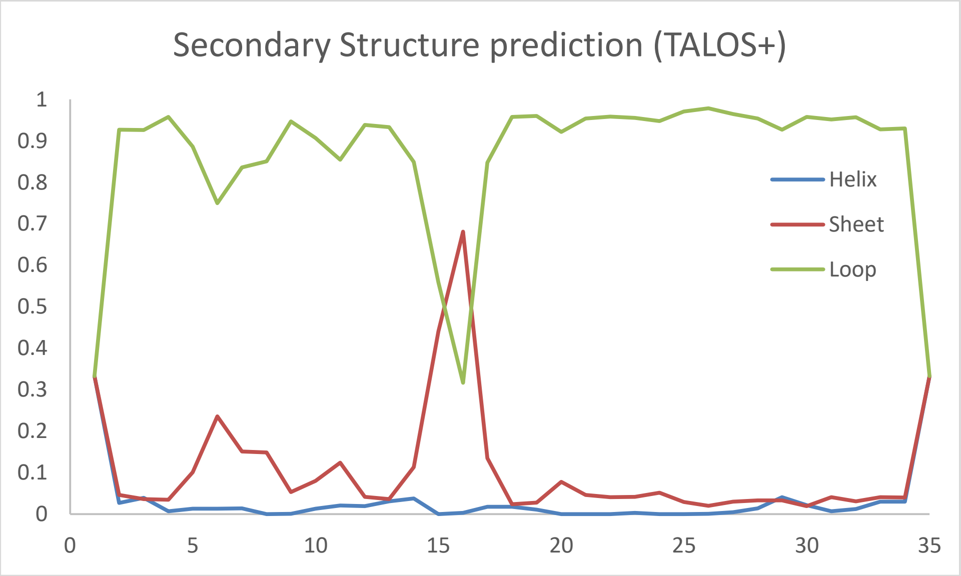

Figure S7. TALOS+ predicted secondary structure of turgencin B_{Mox2} based on all available chemical shifts (HN, N, C, CA, CB, HA, HB). Overall patch of secondary fold is predicted. One residue (C16) is predicted as β-sheet with low confidence, however this is likely an effect of the disulfide bond affecting the chemical shifts.

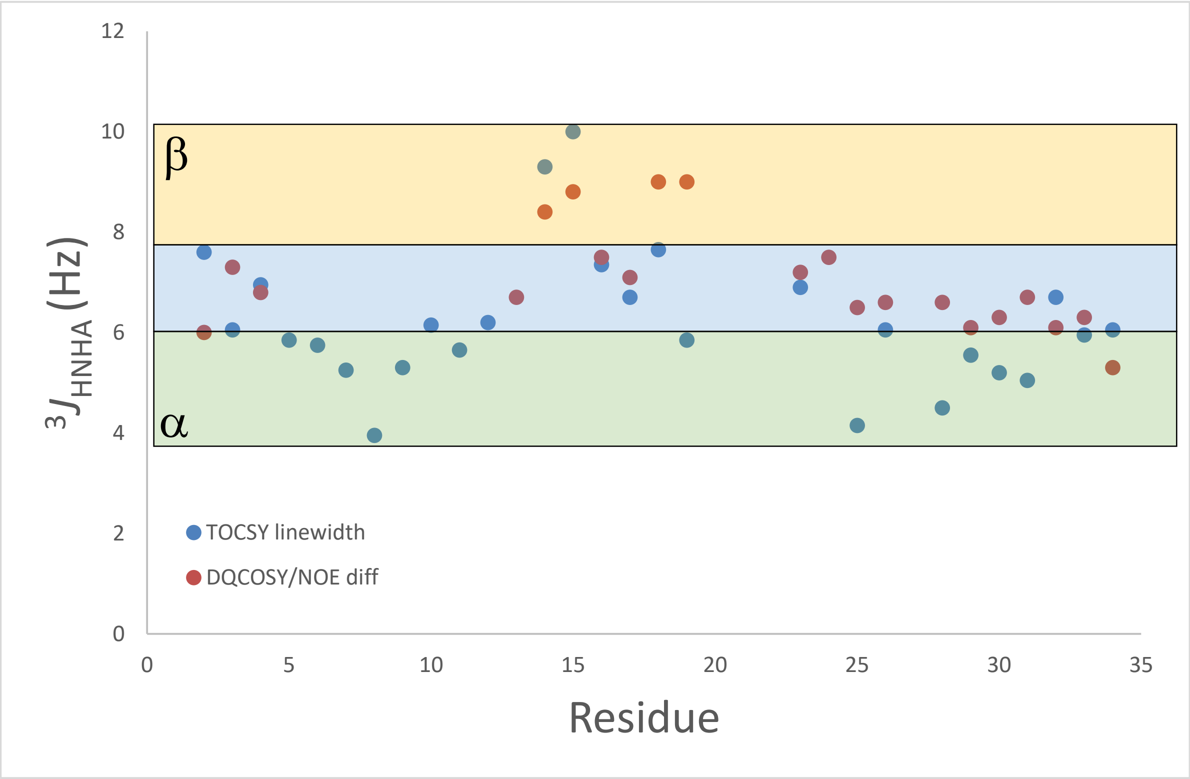

Figure S8. The $^3J_{HNHA}$ coupling constants for turgencin B_{Mox2} structure estimated using two methods using the TOCSY line widths and the sum/diff displacement of DQF-COSY and NOESY slices. The results indicate access to helical structures (green area) for both sides of the turn, but the couplings also suggest significant conformational averaging (blue area).

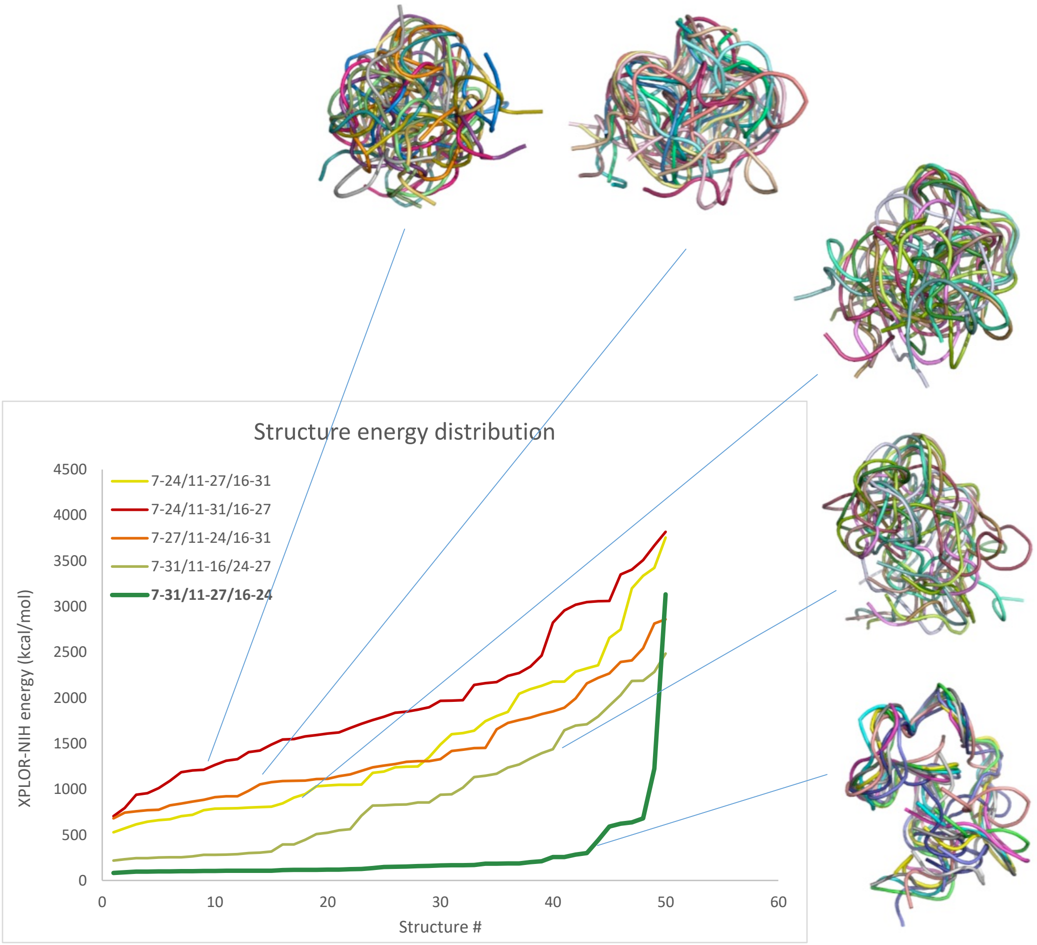

Figure S9. Early test calculations of turgencin B$_{Mox2}$ using crude constraints to evaluate different disulfide patterns using crude constraints. 10 out of 50 structures of each simulation are superimposed, picked at even intervals from the energy profile.

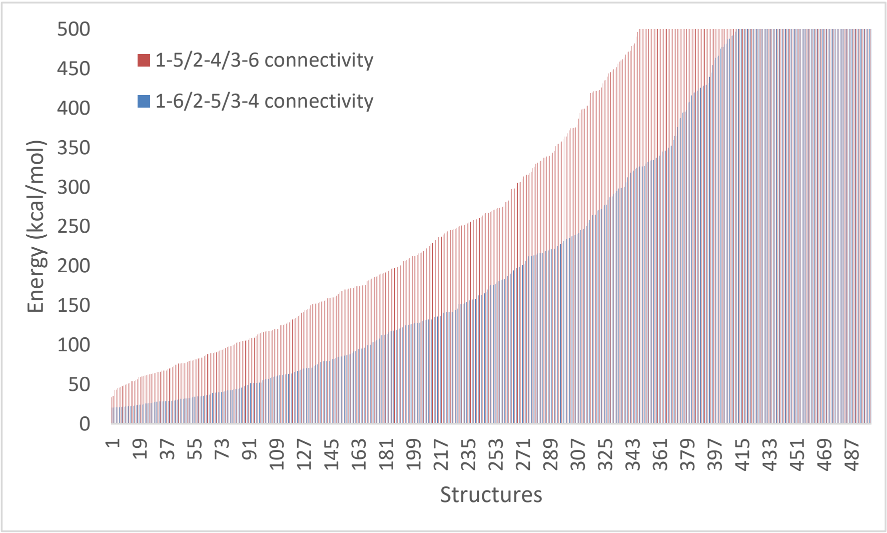

Figure S10. The most viable alternative disulfide pattern of turgencin B$_{Mox2}$ compared to the found pattern in terms of energy of 500 calculated simulated annealing structures using the final constraints.

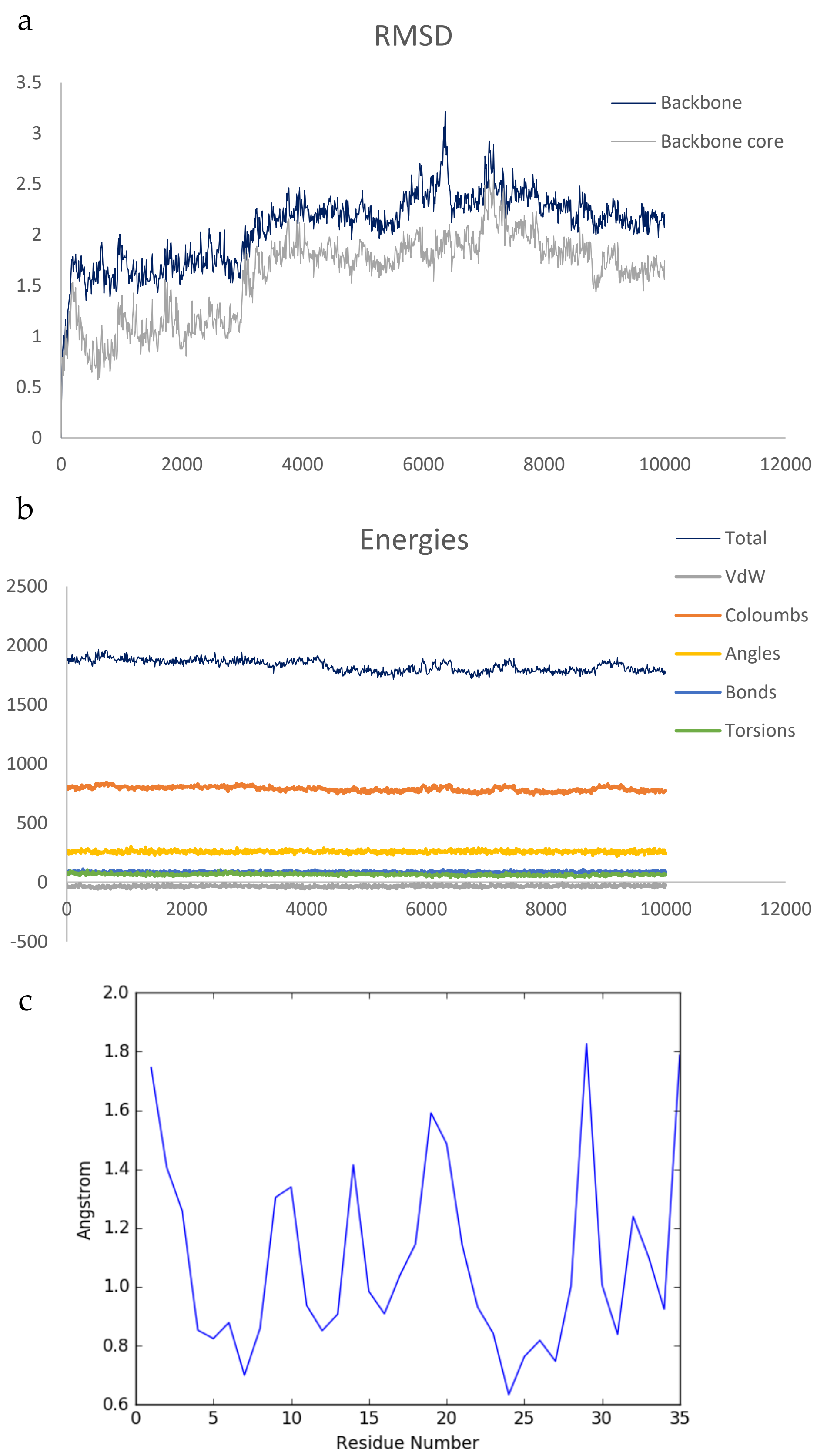

Figure S11. The (a) RMSD relative to the starting frame, (b) energies of turgencin B_{Mox2} and (c) the RMSF of the backbone during the free MD trajectory.

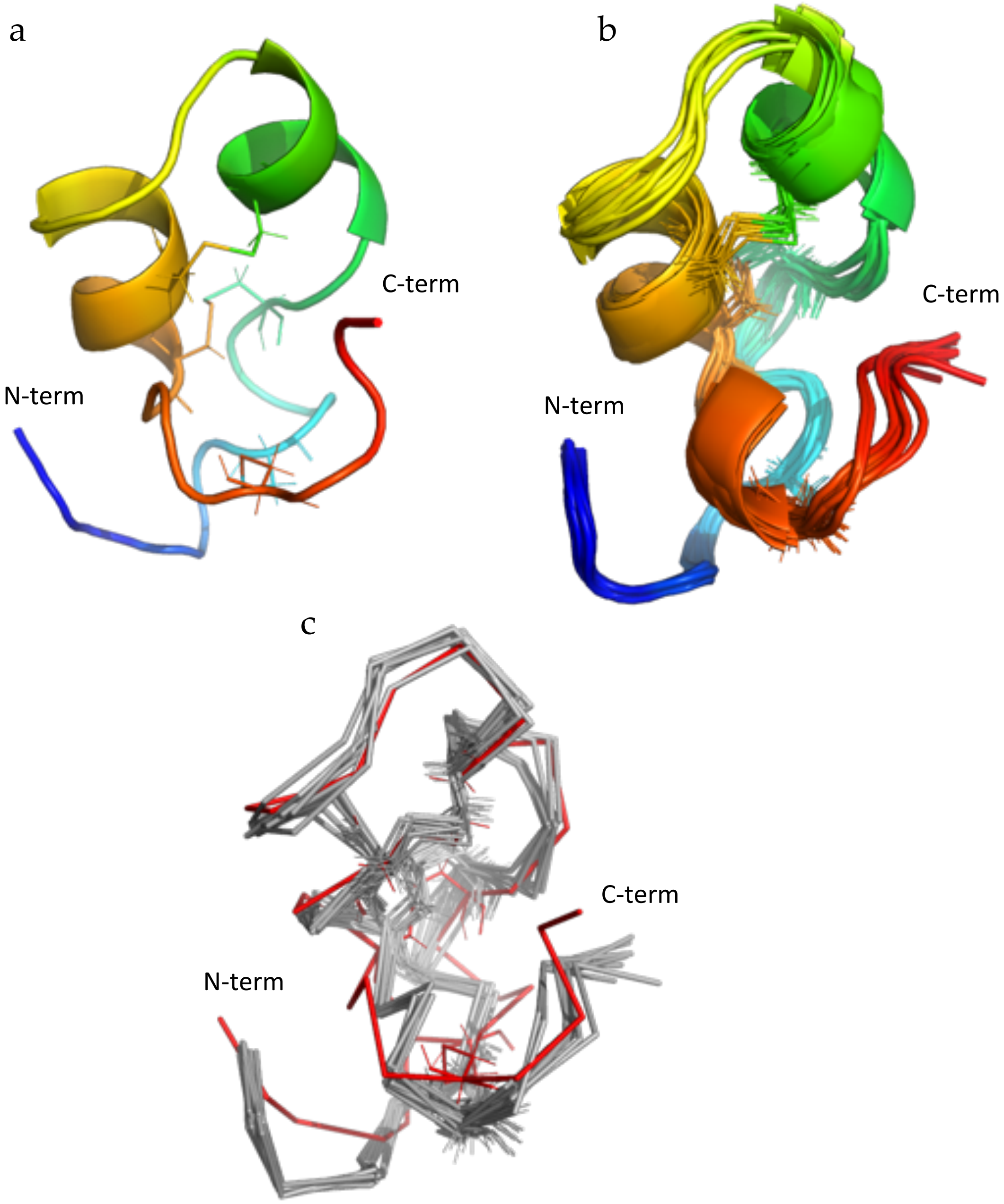

Figure S12. Comparison of NMR and molecular dynamics structures for turgencin B_{Mox2}. (a) The representative NMR structure of turgencin B_{Mox2} selected for MD simulations, and (b) an ensemble sampling the last nanosecond of the simulation. Backbone core (res 6-32) RMSD ~ 1.5 Å. (c) The NMR and MD structures superimposed and displayed with ribbons.

Table S1. Antimicrobial activity of solid phase extract (SPE) fractions and the organic extract. Bacterial test strains: *C. g. - Corynebacterium glutamicum, B. s. - Bacillus subtilis, S. a. - Staphylococcus aureus, E. c. - Escherichia coli, P. a. - Pseudomonas aeruginosa.*

	**Antimicrobial activity (MIC; mg/mL)**				
**Extract**	***C. g.***	***B. s.***	***S. a.***	***E. c.***	***P. a.***
10% MeCN SPE	1.25	5.00	5.00	10.00	5.00
20% MeCN SPE	2.50	2.50	5.00	5.00	5.00
30% MeCN SPE	0.16	0.16	2.50	5.00	5.00
40% MeCN SPE	0.04	0.08	2.50	5.00	2.50
80% MeCN SPE	0.31	0.31	2.50	5.00	2.50
Organic	2.50	2.50	10.00	>10.00	>10.00

Table S2. Calculated and measured monoisotopic *m/z* $[M+4H]^{4+}$ ions of turgencin A, turgencin B, and their oxidized derivatives.

**Peptide**	**Calculated monoisotopic mass $[M+4H]^{4+}$**	**Measured monoisotopic mass $[M+4H]^{4+}$**	**Error (ppm)**
Turgencin A	923.4568	923.4574	0.65
Turgencin A_{Mox1}	927.4555	927.4562	0.71
Turgencin B	885.6526	885.6536	1.11
Turgencin B_{Mox1}	889.6529	889.6532	0.34
Turgencin B_{Mox2}	893.6501	893.6518	1.92

Table S3. Proton chemical shift assignments for turgencin A_{Mox1} in water.

Residue	H (ppm)	Hα(ppm)	Hβ (ppm)	Hγ(ppm)	Others (ppm)
**GLY***1*	8.56	3.91/3.92			
**PRO***2*		4.38	2.24/1.84	2.00/1.94	α:3.50/3.52
**LYS***3*	8.61	4.32	1.80/1.70	1.39/1.43	α:1.57/1.58, α:2.90
**THR***4*	7.62	4.34	4.41	1.18	
**LYS***5*	8.68	3.97	1.82/1.74	1.38/1.40	α:1.66, α:2.94
**ALA***6*	8.34	3.99	1.34		
**ALA***7*	8.00	4.12	1.39		
**CYS***8*	8.16	4.18	3.39/2.96		
**LYS***9*	8.58	3.66	1.80/1.69	1.23/1.22	α:1.58/1.56, α:2.90
**MET***10*	8.16	4.12	2.25	2.94/2.86	α: 1.57, α: 2.62
**ALA***11*	7.96	4.14	1.49		
**CYS***12*	8.10	4.45	3.06/3.12		
**LYS***13*	8.08	3.72	1.99/1.88	1.29	α:1.55, α:2.86
**LEU***14*	7.63	4.07	1.70/1.55	1.68	α1:0.78, α2:0.83
**ALA***15*	7.77	4.31	1.41		
**THR***16*	7.77	4.39	4.06	1.04	
**CYS***17*	8.29	4.21	3.21/2.81		
**GLY***18*	8.43	3.77/3.74			
**LYS***19*	7.80	4.26	1.81/1.67	1.36/1.41	α:1.59, α:2.91
**LYS***20*	7.70	4.75	1.75/1.64	1.26	α:1.63, α:2.90
**PRO***21*		4.74	1.89/2.38	1.99/1.90	α:3.58/3.34
**GLY***22*	8.46	3.67/4.01			
**GLY***23*	7.64	3.76/4.15			
**TRP***24*	8.42	4.34	3.36/3.18		α1:7.26, α1:10.13, α3:7.43, α2:7.13, α2: 7.41,αα3: 7.02
**LYS***25*	8.02	3.54	1.42/1.26	0.89/0.84	α:1.40, α:2.80
**CYS***26*	4.17	3.01/2.97			
**LYS***27*	7.78	3.96	1.74/1.80	1.37/1.29	α:1.54, α:2.84
**LEU***28*	8.09	3.92	1.46/1.41	1.44	α:0.78/0.74
**CYS***29*	7.77	4.15	3.34/2.98		
**GLU***30*	8.43	3.69	2.06/1.88	1.90/2.60	
**LEU***31*	8.22	4.12	1.72/1.55	1.68	α:0.82/0.81
**GLY***32*	7.84	3.86/3.85			
**CYS***33*	7.54	4.66	3.07/2.97		
**ASP***34*	7.74	4.47	2.68		
**ALA***35*	7.54	4.27	1.40		
**VAL***36*	7.53	3.98	2.08	γ1:0.92, γ2:0.85	

Table S4. Carbon chemical shift assignments for turgencin A_{Mox1} in water.

Residue	N (ppm)	Cα(ppm)	Cβ (ppm)	Cγ(ppm)	Others (ppm)
**GLY***1*	109.48	40.56			
**PRO***2*		60.50	29.59	24.34	α:46.89
**LYS***3*	121.19	53.92	30.14	22.24	α:26.21, α:39.33
**THR***4*	111.99	58.48	68.09	19.07	
**LYS***5*	123.81	57.34	29.02	22.26	α:26.72, α:39.32
**ALA***6*	120.98	52.30	15.32		
**ALA***7*	121.95	52.09	15.47		
**CYS***8*	121.10	57.05	35.14		
**LYS***9*	121.30	57.72	30.13	24.40	α:26.28, α:39.30
**MET***10*	120.45	52.02	23.24	48.40	α:26.3, α:36.80
**ALA***11*	123.40	52.42	14.53		
**CYS***12*	116.13	54.33	35.66		
**LYS***13*	123.81	56.97	29.22	22.05	α:26.27,αα:39.18
**LEU***14*	118.52	53.97	39.30	24.20	α1:20.19, α2:21.16
**ALA***15*	120.99	50.92	16.46		
**THR***16*	109.91	61.20	66.78	18.18	
**CYS***17*	119.35	55.29	40.47		
**GLY***18*	118.81	43.86			
**LYS***19*	118.69	54.01	30.64	22.21	α:26.29,αα:39.33
**LYS***20*	117.80	50.79	30.37	22.04	α:26.25, α:39.34
**PRO***21*		61.75	28.76	24.43	α:47.84
**GLY***22*	106.81	42.12			
**GLY***23*	106.85	42.17			
**TRP***24*	122.69	57.01	25.95	108.03	α1:124.59, α2:126.89, α2:126.20, α3:117.93, α2:121.91, α2:112.01, α3:119,26, Nα1:129.22
**LYS***25*	119.54	56.94	28.99	22.57	α:26.31, α:39.15
**CYS***26*	118.96	55.75	36.82		
**LYS***27*	120.16	56.37	29.55	22.18	α:26.125, α:39.22
**LEU***28*	118.33	54.77	39.04	23.94	α:21.82/20.81
**CYS***29*	119.87	52.41	35.34		
**GLU***30*	107.90	57.71	26.49	34.96	
**LEU***31*	121.21	54.92	39.28	24.20	α:22.07

**GLY**32	107.37	43.87			
**CYS**33	117.92	56.11	33.55		
**ASP**34	119.77	53.49	37.87		
**ALA**35	121.58	50.23	16.27		
**VAL**36	117.53	59.61	29.45	γ1:17.82, γ2:18.46	

Table S5. Proton chemical shift assignments for turgencin B_{Mox2} in water.

Residue	H (ppm)	Hα(ppm)	Hβ (ppm)	Hγ(ppm)	Others (ppm)
**GLY**1	7.63	3.65, 3.77	-	-	-
**ILE**2	8.65	3.94	1.79	1.19, 1.42, CH_3: 0.86	δCH_3: 0.81
**LYS**3	8.43	3.88	1.67, 1.76	1.32, 1.47	δCH_2:1.60, εCH_2: 2.89
**GLU**4	8.41	3.82	1.94, 1.98	2.17, 2.25	-
**MET**5	8.01	4.20	2.21, 2.28	2.84, 3.01	εCH_3: 2.58
**LEU**6	8.50	3.97	1.420, 1.749	1.74	δCH_3: 0.75, 0.77
**CYS**7	7,83	4.20	2.820, 3.254	-	-
**ASN**8	8.57	4.20	2.701, 2.748	-	-
**MET**9	8.22	4.14	2.24	2.73, 3.02	εCH_3: 2.57
**ALA**10	7.86	4.21	1.48	-	-
**CYS**11	8.01	4.27	3.04, 3.07	-	-
**ALA**12	8.22	3.73	1.42	-	-
**GLN**13	7.55	4.19	2.06, 2.20	2.40, 2.48	εNH_2: 6.85, 7.31
**THR**14	7.43	4.50	4.26	1.16	-
**VAL**15	8.81	4.09	2.09	0.98, 1.04	-
**CYS**16	8.13	4.89	2.76, 3.31	-	-
**LYS**17	7.23	3.99	1.70	1.30	δCH_2:1.60, εCH_2: 2.90
**LYS**18	8.13	4.17	1.70, 1.80	1.31	δCH_2:1.60, εCH_2: 2.89
**SER**19	7.77	4.37	3.76, 3.83	-	-
**GLY**20	8.18	3.82, 4.12	-	-	-
**GLY**21	8.62	3.85, 4.44	-	-	-

**PRO***22*	-	4.26	1.87, 2.30	1.90, 1.98	δCH_2: 3.54, 3.68
**LEU***23*	8.46	4.09	1.34, 1.70	1.60	δCH_3: 0.80, 0.84
**CYS***24*	7.50	4.19	3.16, 3.21	-	-
**ASP***25*	8.39	4.25	2.62, 2.67	-	-
**THR***26*	8.34	3.86	4.08	1.17	-
**CYS***27*	7.56	4.16	3.01, 3.36	-	-
**GLN***28*	8.29	3.93	1.99, 2.02	2.22, 2.61	-
**ALA***29*	8.38	4.02	1.41	-	-
**ALA***30*	7.64	4.15	1.46	-	-
**CYS***31*	7.49	4.55	3.00, 3.08	-	-
**LYS***32*	7.63	4.03	1.79, 1.85	1.36, 1.49	δCH_2:1.59, εCH_2: 2.86
**ALA***33*	7.57	4.21	1.37	-	-
**LEU***34*	7.61	4.15	1.45, 1.75	1.78	δCH_3: 0.78, 0.82
**GLY***35*	7.93	3.78, 3.87	-	-	terminal-NH_2: 7.05, 7.21

Table S6. Carbon chemical shift assignments for turgencin B_{Mox2} in water.

Residue	N (ppm)	Cα(ppm)	Cβ (ppm)	Cγ(ppm)	Others (ppm)
**GLY***1*	-	61.014	-	-	-
**ILE***2*	122.15	60.82	35.45	14.68, 25.64	δCH_3: 10.32
**LYS***3*	120.82	57.24	29.27	22.65	δCH_2: 26.37 εCH_2: 39.28
**GLU***4*	120.90	57.43	26.15	33.18	-
**MET***5*	118.83	55.76	23.13	48.73	εCH_3: 36.56
**LEU***6*	121.07	55.31	38.68	23.92	δCH_3: 19.85, 22.68
**CYS***7*	121.22	57.19	32.11	-	-
**ASN***8*	120.64	54.04	35.91	-	-
**MET***9*	120.49	55.60	22.93	47.84	εCH_3: 36.547
**ALA***10*	122.84	52.64	14.45	-	-
**CYS***11*	116.93	54.80	35.20	-	-
**ALA***12*	123.91	52.69	15.50	-	-
**GLN***13*	111.92	53.47	27.05	31.25	δCO: 177.40
**THR***14*	108.63	57.095	67.66	18.38	-
**VAL***15*	118.30	61.24	29.56	17.42, 19.53	-
**CYS***16*	116.07	52.91	41.12	-	-

**LYS***17*	120.89	56.09	30.06	22.133	δCH_2: 26.35 εCH_2: 39.32
**LYS***18*	117.66	54.22	29.95	22.18	δCH_2: 26.34 εCH_2: 39.29
**SER***19*	113.19	55.42	61.55	-	-
**GLY***20*	108.86	42.22		-	-
**GLY***21*	110.51	42.04		-	-
**PRO***22*	-	62.77	29.46	24.59	δCH_2: 47.00
**LEU***23*	117.91	55.08	38.44	24.45	δCH_3: 20.31, 22.08
**CYS***24*	119.23	55.88	36.38	-	-
**ASP***25*	120.78	54.79	36.90	-	-
**THR***26*	116.55	63.82	66.02	18.78	-
**CYS***27*	123.34	58.41	35.90	-	-
**GLN***28*	116.81	56.55	25.79	32.02	-
**ALA***29*	123.12	52.24	15.11	-	-
**ALA***30*	121.61	51.88	14.65	-	-
**CYS***31*	115.49	52.02	32.63	-	-
**LYS***32*	119.51	55.41	29.82	22.31	δCH_2: 26.24, εCH_2: 39.20
**ALA***33*	121.34	50.37	15.71	-	-
**LEU***34*	118.72	53.19	39.37	23.84	δCH_3: 20.26, 23.08
**GLY***35*	108.32	42.36	-	-	-

Table S7. Peptides sharing the same disulfide connectivity as turgencin B (C1-C6/C2-C5/C3-C4).

AMP	#aa	Sequence and protein data bank (PDB) ID	Species	Ref.
Turgencin B	35	GIKEMLCNMACAQTVCKKSGGPLCDTCQAACKAL-NH_2	*Synoicum turgens* (ascidian)	This work
TEWP	36	pEKKCPGRCTLKCGKHERPTLPYNCGKYICCVPVKVK (PDB: 2B5B)	*Caretta caretta* (Loggerhead sea turtle)	[1]
Pelovaterin	42	DDTPSSRCGSGGWGPCLPIVDLLCIVHVTVGCSGGFG CCRIG (PDB: 2JR3)	*Pelodiscus sinensis* (Chinese softshell turtle)	[2]
Caenopore-5	81	RSALSCQMCELVVKKYEGSADKDANVIKKDFDAECK KLFHTIPFGTRECDHYVNSKVDPIIHELEGGTAPKDVC TKLNECP (PDB: 2JS9)	*Caenorhabditis elegans* (nematode)	[3]
NK-lysin	78	GLICESCRKIIQKLEDMVGPQPNEDTVTQAASRVCDK MKILRGVCKKIMRTFLRRISKDILTGKKPQAICVDIKIC KE (PDB: 1NKL)	*Sus scrofa* (pig)	[4]
RTD-1	18	GFCRCLCRRGVCRCICTR (cyclic, 3 DSB + head to tail peptide bonds) (PDB: 2LYF)	*Macaca mulatta* (Rhesus monkey)	[5], [6]
Viscotoxin A3	46	KSCCPNTTGRNIYNACRLTGAPRPTCAKLSGCKIISGST CPSDYPK (PDB: 1ED0)	*Viscum album* (mistletoe)	[7] (sequence), [8] (activity)

References

1. Chattopadhyay, S.; Sinha, N. K.; Banerjee, S.; Roy, D.; Chattopadhyay, D.; Roy, S., Small cationic protein from a marine turtle has β-defensin-like fold and antibacterial and antiviral activity. *Proteins* **2006,** 64, 524-531.
2. Lakshminarayanan, R.; Vivekanandan, S.; Samy, R. P.; Banerjee, Y.; Chi-Jin, E. O.; Teo, K. W.; Jois, S. D. S.; Kini, R. M.; Valiyaveettil, S., Structure, self-assembly, and dual role of a β-defensin-like peptide from the Chinese soft-shelled turtle eggshell matrix. *J. Am. Chem. Soc.* **2008,** 130, 4660-4668.
3. Mysliwy, J.; Dingley, A. J.; Stanisak, M.; Jung, S.; Lorenzen, I.; Roeder, T.; Leippe, M.; Grötzinger, J., Caenopore-5: The three-dimensional structure of an antimicrobial protein from *Caenorhabditis elegans*. *Developmental & Comparative Immunology* **2010,** 34, 323-330.
4. Liepinsh, E.; Andersson, M.; Ruysschaert, J.-M.; Otting, G., Saposin fold revealed by the NMR structure of NK-lysin. *Nature Structural Biology* **1997,** 4, 793-795.
5. Tang, Y.-Q.; Yuan, J.; Ösapay, G.; Ösapay, K.; Tran, D.; Miller, C. J.; Ouellette, A. J.; Selsted, M. E., A cyclic antimicrobial peptide produced in primate leukocytes by the ligation of two truncated α-defensins. *Science* **1999,** 286, 498-502.
6. Selsted, M. E., θ-defensins: Cyclic antimicrobial peptides produced by binary ligation of truncated α-defensins. *Current Protein & Peptide Science* **2004,** 5, 365-371.
7. Romagnoli, S.; Ugolini, R.; Fogolari, F.; Schaller, G.; Urech, K.; Giannattasio, M.; Ragona, L.; Molinari, H., NMR structural determination of viscotoxin A3 from *Viscum album L. Biochem. J.* **2000,** 350, 569-577.
8. Giudici, A. M.; Regente, M. C.; Villalaín, J.; Pfüller, K.; Pfüller, U.; De La Canal, L., Mistletoe viscotoxins induce membrane permeabilization and spore death in phytopathogenic fungi. *Physiologia Plantarum* **2004,** 121, 2-7.
